# Comprehensive molecular evaluation of the histone methyltransferase gene family and their important roles in two-line hybrid wheat

**DOI:** 10.1186/s12870-022-03639-0

**Published:** 2022-06-13

**Authors:** Renwei Sun, Jie Gong, Yongjie Liu, Zhaobo Chen, Fengting Zhang, Jiangang Gao, Junmei Cao, Xianchao Chen, Shengquan Zhang, Changping Zhao, Shiqing Gao

**Affiliations:** 1 Institute of Hybrid Wheat, Beijing Academy of Agriculture and Forestry Sciences (BAAFS), Beijing, 100097 China; 2 The Municipal Key Laboratory of the Molecular Genetics of Hybrid Wheat, Beijing, 100097 China; 3 Institute of Grain Crops, Xinjiang Academy of Agricultural Sciences, Urumqi, 830091 China

**Keywords:** Hybrid wheat, Histone methyltransferase, Phylogenetic analysis, Gene expression, *TaCCA1*, Heterosis

## Abstract

**Background:**

Histone methylation usually plays important roles in plant development through post-translational regulation and may provide a new visual field for heterosis. The histone methyltransferase gene family has been identified in various plants, but its members and functions in hybrid wheat related in heterosis is poorly studied.

**Results:**

In this study, 175 histone methyltransferase (*HMT*) genes were identified in wheat, including 152 histone lysine methyltransferase (*HKMT*) genes and 23 protein arginine methyltransferase (*PRMT*) genes. Gene structure analysis, physicochemical properties and subcellular localization predictions of the proteins, exhibited the adequate complexity of this gene family. As an allohexaploid species, the number of the genes (seven HKMTs orthologous groups and four PRMTs orthologous groups) in wheat were about three times than those in diploids and showed certain degrees of conservation, while only a small number of subfamilies such as ASH-like and Su-(var) subfamilies have expanded their members. Transcriptome analysis showed that *HMT* genes were mainly expressed in the reproductive organs. Expression analysis showed that some *TaHMT* genes with different trends in various hybrid combinations may be regulated by lncRNAs with similar expression trends. Pearson correlation analysis of the expression of *TaHMT* genes and two yield traits indicated that four DEGs may participate in the yield heterosis of two-line hybrid wheat. ChIP-qPCR results showed that the histone modifications (H3K4me3, H3K36me3 and H3K9ac) enriched in promoter regions of three *TaCCA1* genes which are homologous to *Arabidopsis* heterosis-related *CCA1/LHY* genes. The higher expression levels of *TaCCA1* in F_1_ than its parents are positive with these histone modifications. These results showed that histone modifications may play important roles in wheat heterosis.

**Conclusions:**

Our study identified characteristics of the histone methyltransferase gene family and enhances the understanding of the evolution and function of these members in allohexaploid wheat. The causes of heterosis of two-line hybrid wheat were partially explained from the perspective of histone modifications.

**Supplementary Information:**

The online version contains supplementary material available at 10.1186/s12870-022-03639-0.

## Background

Common wheat (*Triticum aestivum* L.), which is among the most widely consumed foods globally, is grown all over the world, second only to rice (*Oryza sativa* L.). It is generally believed that wheat was domesticated in Western Asia about 8000 years ago and consists of allohexaploid (AABBDD) in three sets of genomes: *Triticum urartu* (A-genome donor), an *Aegilops speltoides*-related grass (B-genome donor), and *Aegilops tauschii* (D-genome donor) [1, 2]. The huge genome and complex structure of wheat kept its genetic information a mystery for a long time. After years of unremitting scientific effort, some high-quality genome annotation data have been released, and multiple genome assemblies are available, which provide a powerful basis for the study of functional genomics [3–7]. A large amount of genetic diversity of cultivated crop species reside in its wild relatives. To harness that diversity and to expand the genetic base of the cultivated species, it is important to understand the evolutionary relationships among them and to know which genomic and phenomic tools would be appropriate.

Epigenetics, including DNA methylation, chromatin modification, and RNA editing, is a branch of genetics that studies the heritable changes of gene expression without changing the nucleotide sequence of genes [8]. In eukaryotes, nucleosomes that contain four groups of proteins (H2A, H2B, H3, and H4) are the main structural components of chromatin. The four groups of proteins are two H3:H4 dimers and two H2A:H2B dimers, as well as a mixture of 146 bp DNA and a double helix. The N-end trailing of each histone conserved in vitro is the point of many targeted signal transduction pathways, resulting in post-translational modifications [9, 10]. Histone methylation is one of most important epigenetic modifications, and it plays a fundamental role in a range of biological processes, from transcriptional regulation to heterochromatin formation [11]. Methylation sites are usually located on the lysine (K) and arginine (R) residues of H3 and H4 and thus determine the two methylation patterns of histone lysine (Lys, K) methylation (HKMT) and histone arginine (Arg, R) methylation (PRMT). Methylation occurred in the arginine and lysine residues of histones, including seven sites in arginine and 17 sites in lysine [11]. These complex modification processes, one earlier and one more advanced, are deeply associated with gene expression. Studies have shown that histone methylation, catalyzed by HKMT and PRMT proteins, plays an important role in plant developmental processes, such as floral organogenesis, seed development, and plant senescence and defense [12–16].

The two-line method is a preferred way of realizing the heterosis of wheat, but the results of research on the molecular mechanism of the heterosis remain relatively weak. Generally, there is a strong correlation between heterosis and the genetic distance of the parents [17], but the study has shown that genetic distance does not fully explain heterosis [18]. The allelic variation caused by epigenetic modification provides a novel way of explaining heterosis. Previous study has shown that histone modification is closely related to the differential expression of genes in hybrid maize [19]. The methylation of H3K4me2 can reduce the expression of two genes: *CCA1* and *LHY*, which are the cores of heterosis in *Arabidopsis*, resulting in the improvement of photosynthetic efficiency of F_1_ and the emergence of obvious hybrids [20]. Generally, H3K9ac and H3K4me3 occur in regions of normal chromatin and activate the expression of genes, whereas H3K9me2 and H3K27me3 tend to occur in the heterochromatin regions and usually inhibit gene expression [21, 22]. The levels of H3K4me3 and H3K27me3 vary greatly among ecotypes and subspecies, but they showed additive patterns in *Arabidopsis* hybrids, whereas in rice they showed non-additive ones [19, 23]. Proteins in hybrid F_1_, such as H3K9ac, H3K4me2, and H3K4me3, may be related to heterosis through the modification of different types of histone proteins to regulate circadian genes *CCA1* and *LHY* [20]. However, due to the various methods of modification, the regulation mechanisms of histone modification and heterosis require further exploration. lncRNA-mediated deposition of H3K27me3 is critical for chromatin modification and transcriptional regulation [24, 25]. lncRNAs participate in histone modification to regulate a series of important biological processes, such as vernalization and flowering, which can affect plant yield and quality [26, 27]. MicroRNAs (miRNAs) are a class of endogenous small noncoding RNAs which are involved in the regulation of gene expression at the posttranscriptional level by degrading their target mRNAs and/or inhibiting their translation. MicroRNA-mediated regulation is a key component in a wide range of biological processes such as plant developmental plasticity, abiotic/biotic responses, and symbiotic/parasitic interactions [28, 29]. *HMT* genes have been identified and studied in many plant systems and are widely involved in the regulation of plant growth and development [13, 30–36]. Although it is a fundamental process in an important cereal crop, histone methylation remains mysterious, and the role of regulatory factors such as miRNAs and lncRNAs in regulating histone methylation is largely unknown.

In this study, we provided a detailed overview of the phylogeny and expression of *HMT* genes in different hybrid bread wheats. We aim to explore the distribution and evolution of HMT members that catalyze histone methylation in wheat, and search for some specific HMTs which may participate in the construction of heterosis in hybrid wheat. All our efforts enhance the understanding of the evolution and function of the histone methylation family and sheds light on the histone methylation involved in regulating wheat yield.

## Results

### Identification of *TaHMT* genes from the whole wheat genome

**Fig. 1:**
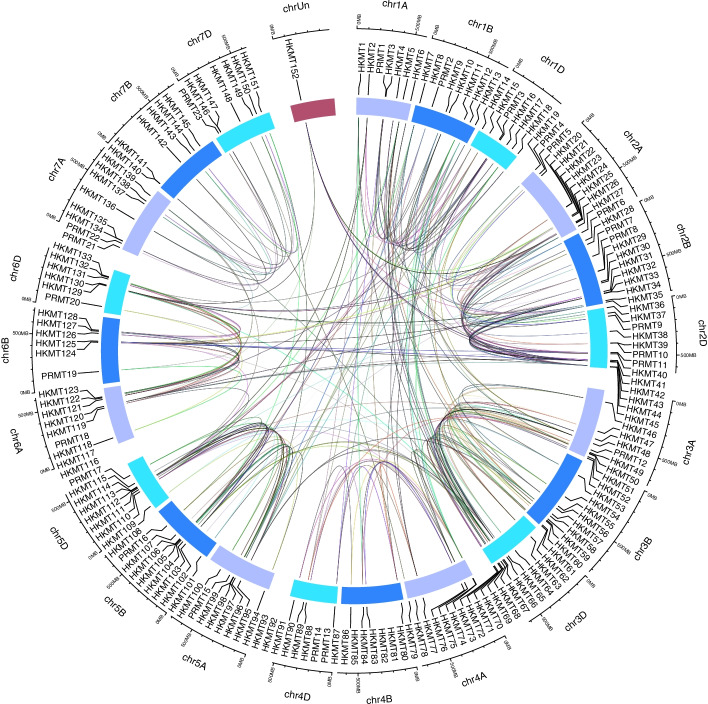
Collinearity analyses of all *TaHMT* genes. All 175 genes were mapped to their respective locus in the wheat genome in a circular diagram. Sub-genomes are indicated by different shades of blue (outer track). Homoeologous genes were inferred by phylogeny (shown in the Materials and Methods section)

Hmmsearch and the BLASTP program obtained 152 *HKMT* (histone lysine methyltransferase) genes and 23 *PRMT* (histone arginine methyltransferase) genes, which were named *TaHMT1*–*TaHMT152*, coinciding with *TaHKMT1*–*TaHKMT152*, *TaHMT153*–*TaHMT175*, and *TaPRMT1-23*, following the nomenclature 1A to 1D, 2A to 2D, up to 7A to 7D, based on their position on the chromosome. In total, we identified 175 genes that catalyze two kinds of histone methyltransferases (Fig. 1, Table 1, Table S1 and Table S2). Chromosome distribution showed that the *HKMT* gene family of wheat was distributed across all chromosomes and showed a trend of cluster distribution on chr2 and chr3. The small number of *PRMT* genes entails that they are orphans that exist on some chromosomes, such as chr4A (Figure S1).

**Table 1:** Numbers of *HMT* genes in wheat and related species

species	Number of *HMT*s	Number of *HKMT*s	Ratio to wheat	Number of *PRMT*s	Ratio to wheat
Wheat	175	152	–	23	–
*Arabidopsis*	57	48	3.17	9	2.56
Rice	47	39	3.90	8	2.88
*Brachypodium*	56	48	3.17	8	2.88
*Sorghum*	54	46	3.30	8	2.88
*Hordeum*	49	42	3.62	7	3.29

### *HMT* gene family of wheat has expanded with more in-paralogs genes

*Sorghum bicolor*, *Hordeum vulgare*, and *Brachypodium distachyon* are usually considered to be relatives of wheat due to the whole-genome duplication events that occurred at about 45–60 million years ago [37]. In this study, the *HMT* gene families in *Arabidopsis thaliana*, *Oryza sativa*, *Hordeum vulgare*, *Sorghum bicolor*, and *Brachypodium distachyon* were also comprehensively identified in Table 1 using the same method as that employed for wheat. According to previous results, the number of *HMT* genes family in diploid species is between 40 and 60. We found that there were 47 and 57 *HMT* genes in rice (*Os*) and *Arabidopsis* (*At*), respectively, which is close to the results previously obtained. In addition, 54, 56, and 49 *HMT* genes were obtained from the *Sorghum bicolor* (*Sb*), *Brachypodium distachyon* (*Bd*), and *Hordeum vulgare* (*Hv*) genome data, respectively. The proportion of gene quantity between wheat and other species was 3.07 (*At*), 3.72 (*Os*), 3.24 (*Sb*), 3.13 (*Bd*), and 3.57 (*Hv*), close to a ratio of 3:1, as expected (χ^2^ test, *P* > 0.05).

To further explore the evolutionary relationship between wheat and the other five species, the orthologs and in-paralogs between each pair of these species were calculated. The number of orthologs in each species with one other genome is shown in Table 2. The result shows that wheat had a high average ortholog group size of 2.32, which also meant that every *HMT* gene had an average of 1.32 paralogs. The group sizes of other five species were significantly smaller than those of wheat, indicating that the genome ploidy of wheat enriches the number of paralogous genes for the *HMT* gene family. Significantly, the fact that wheat and *Brachypodium distachyon* clearly have more orthologous genes proves a later divergence between the two species, which is also a reflection of the previous conclusion that *Brachypodium distachyon* has a common ancestor with wheat.

**Table 2:** Total number of orthologs identified by In-paranoid 4. 1

species	Wheat	*Arabidopsis*	Rice	*Sorghum*	*Brachypodium*	*Hordeum*	Average in-paralogs
Wheat	–	137/401	121/43	132/46	141/50	111/44	2.32^a^
*Arabidopsis*	48/40	–	42/34	42/34	45/36	42/34	1.23
Rice	44/43	38/34	–	40/39	38/37	36/36	1.04
*Sorghum*	47/46	39/34	40/39	–	44/44	40/39	1.04
*Brachypodium*	50/50	44/36	38/37	47/44	–	40/40	1.06
*Hordeum*	44/44	38/34	37/36	40/39	40/40	–	1.03

^a^ The number of orthologs in an organism when clustered with another genome was shown on the left of the slash while the number on the right of the slash referred to the number of ortholog groups between two species. For wheat, average size of ortholog group: 2.32 = (137/40 + 121/43 + 132/46 + 141/50 + 111/44)/5. Notably, Table 2 was not a symmetrical table, since gene duplication frequency in organism ‘A’ generally differed from that in organism ‘B’ since speciation of organism ‘A’ and ‘B’ and thus the number of ‘A’ genes having orthologs in organism ‘B’ was unequal to the number of ‘B’ genes having orthologs in organism ‘A’

According to the published wheat genome data, there are about in homologous groups of three, termed triads (1:1:1; 35.8% of genes) (IWGSC, 2018) [38]. Our results revealed a higher proportion 92.76% of *HKMT*s were in triads. In *PRMT*s, the ratio is 91.3% (Table 3). The high homology retention rate indicates that the *HMT* gene of three sets of wheat chromosome donors exhibited a certain conservation; in other words, there was almost no lack of homologous genes. This kind of conservative combination of triplets and high homology retention rate can also explain the richness of some gene families in wheat, such as the *HMT* genes. In addition to this 1:1:1 correspondence, some genes have expanded in breadth along the chromosomes. These members do not strictly follow the three homologies, like *HKMT1* and *PRMT1*. In addition to a correspondence to genes on chromosomes with A/B/D homology, they also show homology with members of other chromosomes to a certain extent. The homology of these genes may have been established before the formation of wheat polyploidy.

**Table 3:** Groups of homoeologous *HMT* genes in wheat

Homoeologous group (A:B:D)	Number of groups	Number in total	Ratio in the gene family
*HKMT*			
1:1:1	47	141	92.76%
Paired	3	6	3.94%
Orphans/singletons	5	5	3.30%
*PRMT*			
1:1:1	7	21	91.30%
Paired	1	2	8.70%
Orphans/singletons	0	0	0.00%

### Structure, phylogeny, and domain analysis of *HMT* genes in wheat

The analysis of the physical and chemical properties of the protein sequences encoded by 152 *TaHKMT* genes revealed that the amino acid content of different HKMT proteins varied significantly, and the molecular weight of the encoded protein was significantly different from 24.76 kD (*TaHMT145*) to 221.14 kD (*TaHMT118*). Compared to the *HKMT* gene family, the protein encoded by the *PRMT* gene family seems not to show a significant difference in physicochemical properties. In the HKMT protein, the differences in the physicochemical properties of the protein were significantly higher than those of the histone arginine methyltransferase. With the exception of *TaHMT154*, the isoelectric point (pI) of the proteins encoded by the histone arginine methyltransferase gene was less than 7.0. This shows a more consistent acidity that tends to play a role in the acidic subcellular environment, which may be due to the coding of weakly acidic protein by the *PRMT* genes. Only 5.26% (8 /152) of HKMT proteins showed high stability, while 34.78% (8/23) of PRMT members were proteins with high stability. These findings suggest that HKMT may have wider catalytic functions and may be involved in more biological processes.

**Fig. 2:**
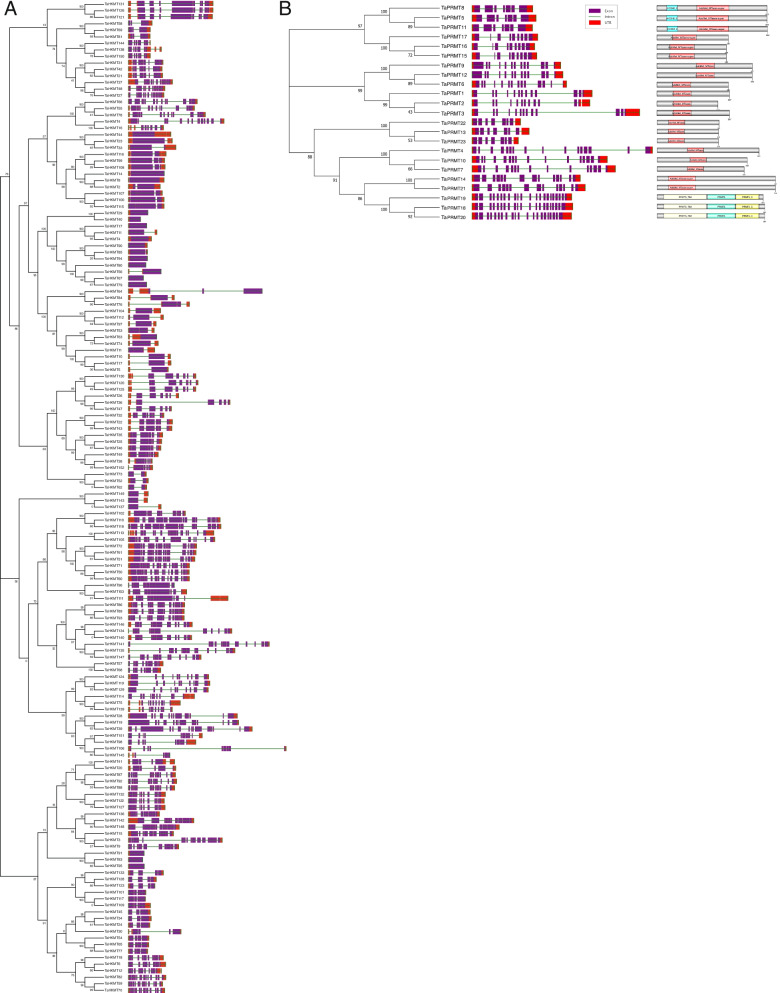
Analysis of phylogeny, structures, and protein motifs of all 175 TaHMTs in wheat. **A** and **B** Gene phylogeny, structures, and protein motifs of 152 *HKMT* genes. **C** Gene phylogeny, structures, and protein motifs of 23 *PRMT* genes. The full-length coding sequence (CDS) were aligned with Clustal X, and the phylogenetic tree was constructed using the Maximum Likelihood method by MEGA-X. In the gene structures, the purple boxes represent exons, and the green lines represent introns. The untranslated regions (UTRs) are indicated by red boxes. The sizes of the exons and introns can be estimated using the scale at the bottom. The protein motifs were drawn by different colored boxes as shown in the figure using Dog 2.0

Interestingly, the subcellular localization prediction analysis told us that most HKMT proteins are located in the nucleus, while most PRMT proteins are located in the cytoplasm (Table S1). This difference in localization also implies that they may have different physiological functions. The structural analysis of all *TaHMT* genes indicated that all of the members contained exons, but the number of them was quite different. The *TaHMT* gene family has a large number of introns and exons, most of which have more than four exons, and their genetic structure is complex and diverse. Interestingly, the number of exons in the *HKMT* gene family presents a huge difference from 25 (*TaHMT113*) to 1 (*TaHMT13*.etc.) (Fig. 2, Table S1).

**Fig. 3:**
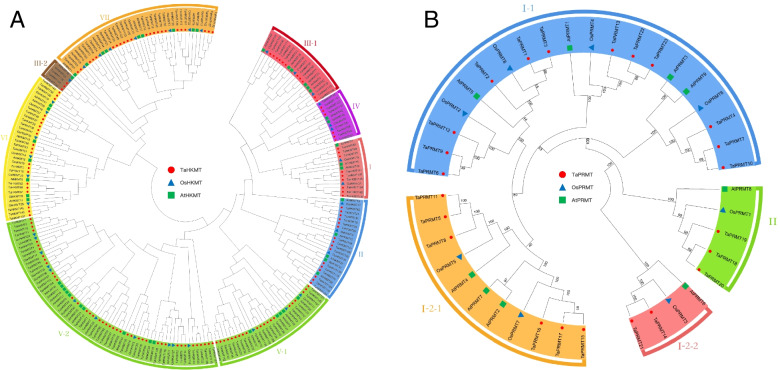
The phylogeny of HKMT (**A**) and PRMT (**B**) proteins from wheat, rice, and *Arabidopsis*. Wheat proteins are colored red and are subclade-specific, whereas rice and *Arabidopsis* genes are in blue and green, respectively. The HKMT proteins were divided into seven subfamilies and PRMT were divided into four subfamilies

All 152 TaHKMT proteins were divided into seven classes according to the classification of the SET gene family in *Arabidopsis* [39]. The seven classes have fully different domain architectures and motif compositions (Figs. 2 and 3). Class I, namely, Enhancer of zeste [E(Z)] homologs (H3K27), has unique domains, such as SWI3, ADA2, N-CoR, and TFIIIB (SANT) DNA-binding domains and DNA binding protein CXC domain. These domains may facilitate the ability of the SET domain to modify histones. Class II consists of 16 SET proteins, which are homologous with *Drosophila* ortholog ASH1 and may lead to H3K4 and H3K36 modifications. Class III is believed to be the SET protein most responsible for the active mark H3K4me1/2/3, similar to the *Drosophila* ortholog TRX.

Class IV is the least of all of the classifications, which is highly conservative, and it is mainly characterized by a PHD finger and a C-terminally located SET domain. It is suggested that the members of this group may be involved in the methylation of H3K4. Class V is the largest population, consisting of 63 *SET* genes homologous to *Drosophila* SU(VAR)3–9, which is divided into two different subgroups. Subgroup I contains the WIYLD domain, pre-SET domains, SET domains, and post-SET domains, while subgroup II contains a typical SRA domain at the N-terminus, playing an essential role in the establishment of heterochromatic mark H3K9me1/2/3, as well as H4K20mes and H3K27me2. Classes VI and VII contain 44 members with interrupted SET domains and Rubis-subs-bind whose functions are to be determined.

**Fig. 4:**
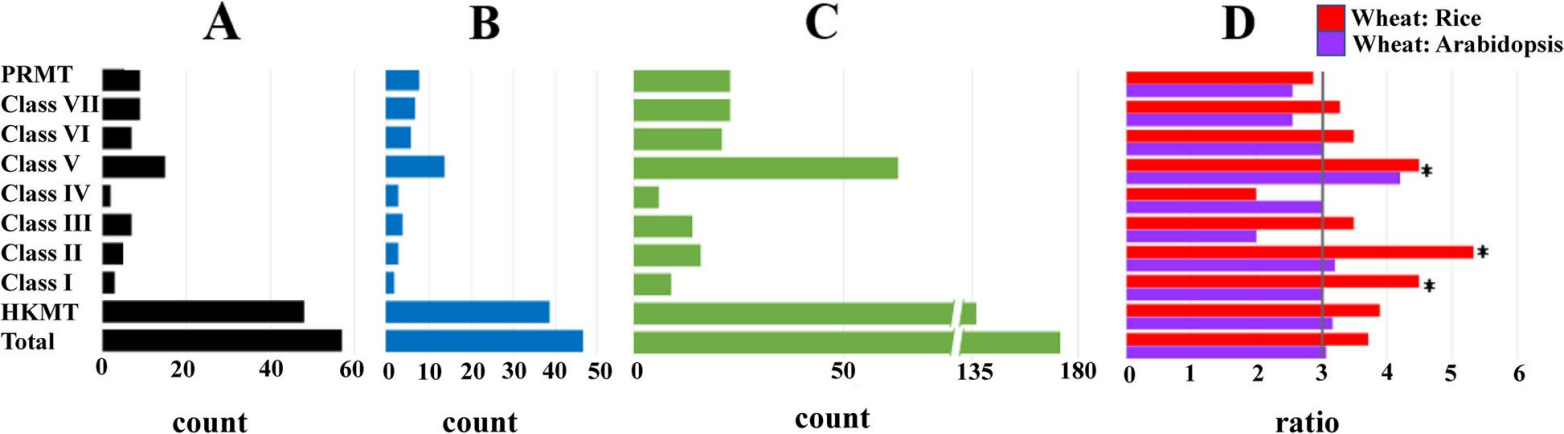
The number of *HMT* genes identified per subfamily in **A** *Arabidopsis*, **B** rice, and **C** wheat. **D** The ratio of total *HMT* gene numbers to those in all subfamilies is shown for wheat: rice (red) and wheat: *Arabidopsis* (purple). In **D** the expected ratio (3:1) is indicated by a black line, and asterisks mark a significant deviation from the expected value using χ^2^ test, *P* < 0.05

The 23 TaPRMT proteins were divided into four classes, following previous opinion [40]. Classes I-1 to I-3 contain 20 members. Most of them include AdoMet-MTases with arginine methyl-transfer activity, which catalyzes the formation of mono-methylarginine and asymmetric dimethylarginine. TaPRMT18 to 20 belong to class II, which owns the core domain PRMT5, which catalyzes mono-methylarginine and symmetric dimethylarginine (Figs. 2C and 3B). The main reason that the *HMT* gene family has a large number of members is that the class I/II/V HKMT subfamily is expanding its members during evolution. The ratio of their number in wheat to that in rice and *Arabidopsis* is higher than the average of 3 (χ^2^ test, *P* < 0.05) (Fig. 4, Table S3).

### Expression characterization and transcription regulation analysis of *TaHMTs*

**Fig. 5:**
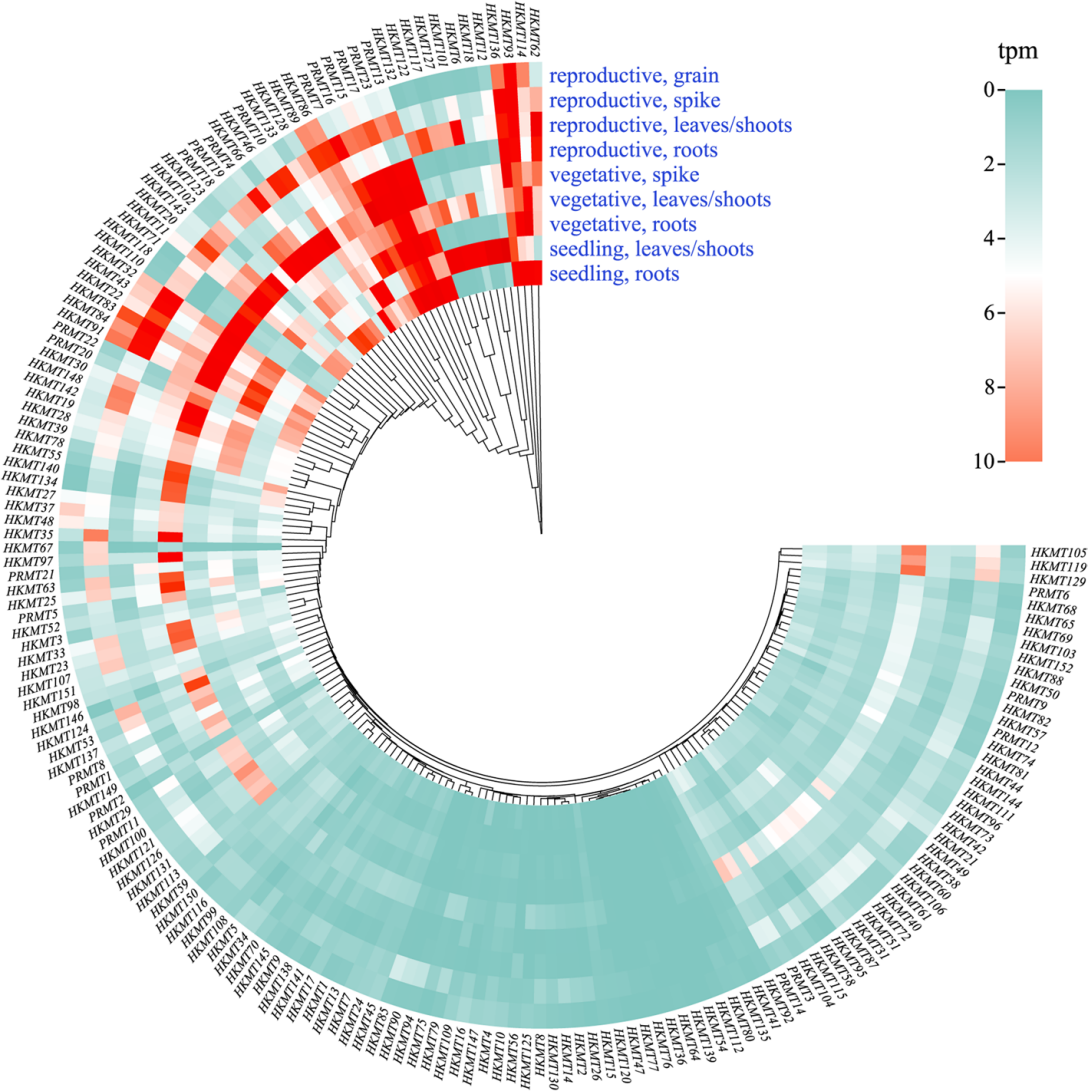
Tissue and organ expression of all identified *HMT* genes. The tpm values of all gene expression data are retrieved from downloaded from wheat-expression.com.. Blue modules indicate low expression level and red modules indicate high expression level. The concentrate of expression was in spike both at vegetative growth and reproductive growth

At present, the expression level of *HMT*s in various wheat tissues without any stress pressure is still unknown. In our study, the expression of *TaHMT* genes in the root, young leaf/shoot, and the young spikes during the vegetative growth stage were significantly higher than those of other tissues at other stages, and they may be widely involved in the physiological activities of wheat growth and development. Spike, as the reproductive organ, exhibits all-round expression for the genes during vegetative and reproductive stages. This result also indicates that the gene expression levels were significantly different in different organs and tissues, which may be biased. For example, *TaHKMT6* and *TaHKMT18* showed obvious tissue specificity, which are characterized by high expression only in the leaves/shoots during the seedling stage, and a relatively low relative expression in young spikes (Fig. 5). There are many inert genes whose expression level is always very low, such as *TaHKMT15* and *TaHKMT41*.

Cis-regulatory elements analysis showed that light responsiveness related elements were the most extensive-elements in the promoter regions of *TaHMT* genes. Except for *TaHMT151*, all genes contained light responsiveness related elements SP1 (GGGCGG) or GT1 motif (GGTTAA), of which 29 *TaHMT*s also contained circadian elements. Most *TaHMT* genes (173/175) contained stress resistance related elements, such as MBS motif related to drought-inducibility, ABRE motif to abscisic acid responsive, LTR motif to low-temperature responsiveness (Figure S2). MicroRNAs (miRNAs) are a class of endogenous small noncoding RNAs which are involved in the regulation of gene expression at the posttranscriptional level by degrading their target mRNAs and/or inhibiting their translation. We found that 11 different miRNAs had 15 targeting sites in body parts of 11 *TaHMT* genes (Table S4, Figure S6). Some miRNAs can act on multiple *TaHMT* genes, and one *TaHMT* gene can also interact with multiple miRNAs. Two miRNAs were found to inhibit expression of the target genes at the translational level: *tae-miR5049-3p* inhibiting gene *TaHMT40* and *tae-miR1134* inhibiting gene *TaHMT172*.

### Expression analysis of *HMT* genes between parents and hybrids

**Fig. 6:**
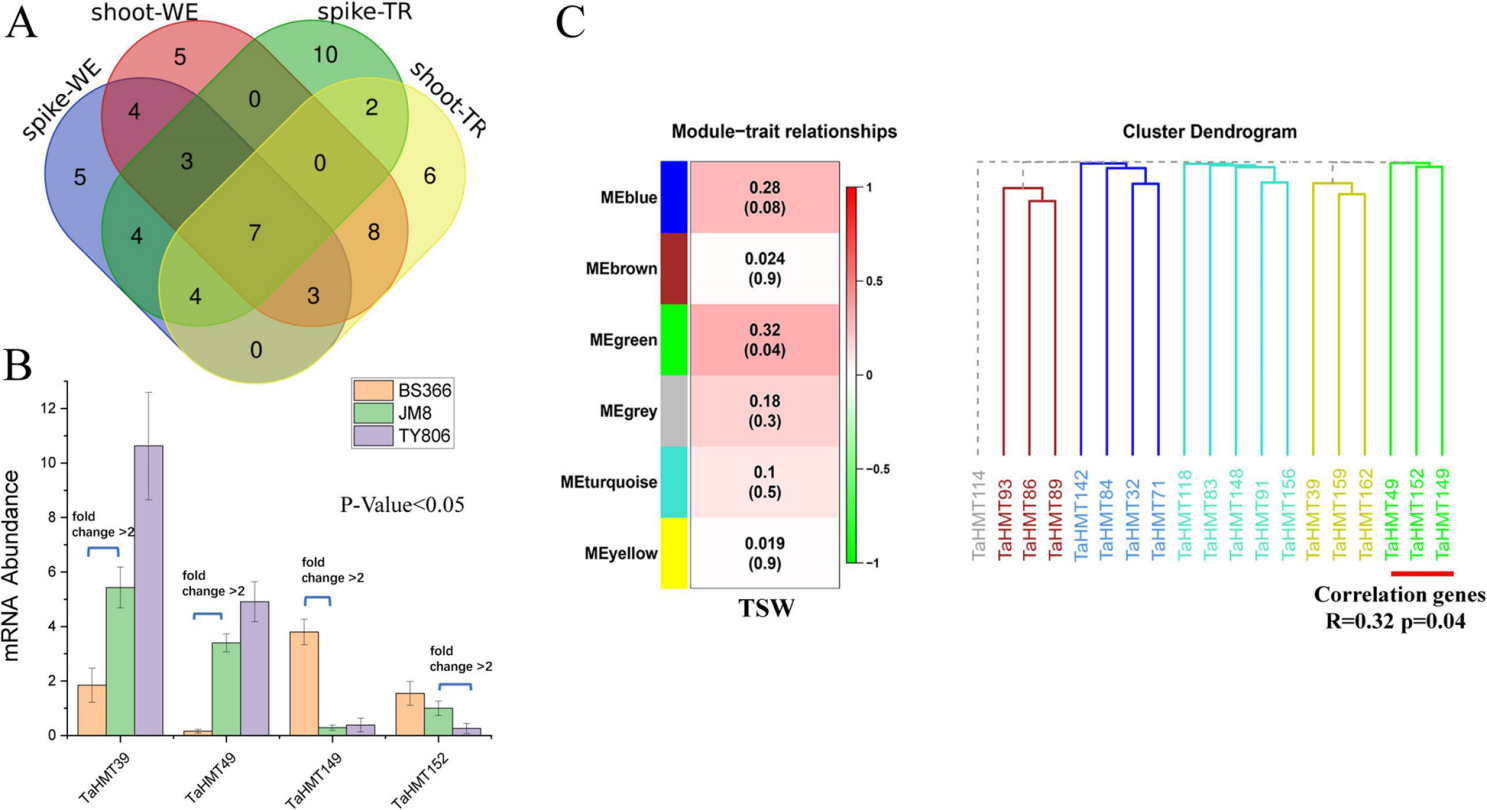
Screening and expression analysis of differentially expressed genes. **A** Pooled analysis of high expression level genes from two databases using Venn diagrams. **B** The mRNA abundance of four genes in JM8 combinations from transcriptome data, presented a significant difference between any one parent and F_1_. The relative expression levels were significantly different, which met the |fold change|> 2 and *p* < 0.05 in spikes. Values are given as means ± SD (*n* = 3). **C** WGCNA analysis of 19 genes in spikes, wherein only three genes show correlations with TSW

Based on our transcriptome sequencing results of JM8 (BS366 × TY806), we obtained four genes whose relative expression level was significantly different (|fold change|> 2, *p* < 0.05) from that of their parents in spikes (Fig. 6B). These parts are *TaHMT39*, *TaHMT49*, *TaHMT149*, and *TaHMT152*, which may be involved in heterosis during the period of wheat reproductive growth in spikes. However, there are no significant DEGs screened in the tillering tissue. We conducted a profound analysis of the transcriptome sequencing data and the expression data downloaded from wheat-expression.com. We defined the transcriptome sequencing data as shoot-TR and spike-TR, respectively, and the data obtained from the website database was defined as shoot-WE and spike-WE, representing two organizations in two periods. The two parts showed the matching degrees of 56.67% in shoot tissue, with 43.33% in spikes (Fig. 6A, Table S5). Our expectation of seven active genes at high levels of expression in the four groups was met. Finally, among them, 12 genes were screened in shoots, and 15 were screened in spikes for further analysis.

### *TaHMT* genes showed multiple gene expression patterns in hybrid combinations

Together with the 1000 seed weight (TSW) and effective tillers per plant in wheat, four *TaHMT* DEGs and 27 genes obtained by the previous methods were analyzed by qRT-PCR. WGCNA was performed on the expression results. The relative expression level of each maternal plant was standardized as relative 1, and all of the expression results were shown in Figure S3.

**Fig. 7:**
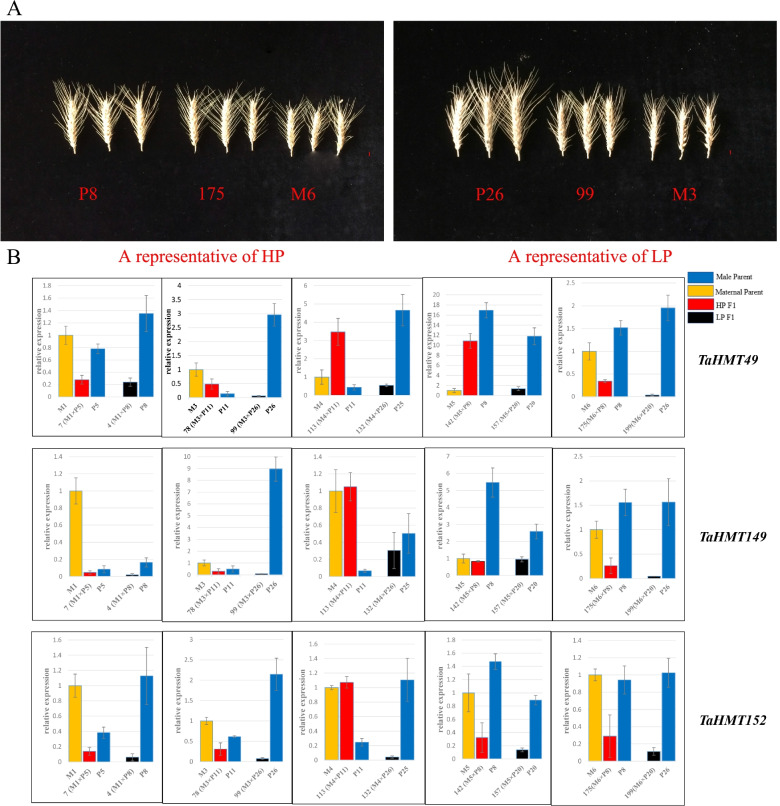
Analysis of three differentially expressed genes in five different two-line hybrid combinations. **A** Combi175, a representative of HP material. Combi99, a representative of LP material. The length of the red ruler represents 1 cm. S **B** Expression levels of three differentially expressed genes in five two-line hybrid combinations. Values are given as means ± SD (*n* = 3)

**Fig. 8:**
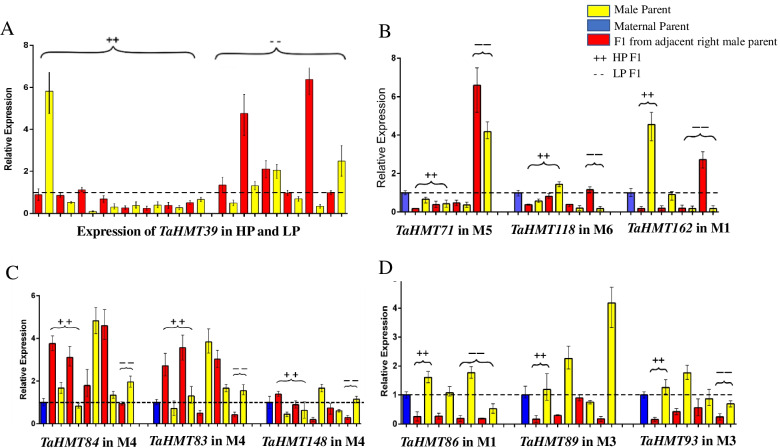
Multiple expression patterns in all combinations. **A** The expression results *TaHMT39* in HP and LP materials. **B** Negative correlation expression patterns of *TaHMT7*1, *TaHMT118*, and *TaHMT162*. **C** Positive correlation expression patterns of *TaHMT83*, *TaHMT84*, and *TaHMT148*. **D** *TaHMT86*, *TaHMT89*, and *TaHMT93* showed under-dominance patterns in M1 and M3 combinations. The relative expression of the maternal parent of each combination was relative 1, which is not shown in Fig. 8**A** but appears in the blue-colored box in Fig. 8**B**–**D**, marked as a dotted line. Red represents the relative expression of F_1_, and the yellow adjacent to the right represents the relative expression of its male paternity. +  + marks applicability of all HP combinations, and – marks LP combinations. Values are given as means ± SD (*n* = 3)

Based on the trait data of TSW, the expression of 19 genes in spikes showed low correlations on the whole. The results were divided into six modules, in which three DEGs (green modules), *TaHMT49*, *TaHMT149*, and *TaHMT152* showed a certain correlation (*R* = 0.32, *p* = 0.04) (Fig. 6C). In general, their expression levels showed a positive correlation with the TSW in the samples. These three genes, on the whole, showed under-dominant expression patterns. In addition, their expression in transgressive materials was somehow higher than that of the low-parent materials corresponding to transcriptome sequencing data (Fig. 7). In the HP combination, the gene expression in F_1_ were up-regulated by about 20 times at most (*TaHMT49* in Combi201), while in some LP combinations, it was down regulated by more than 30 times (*TaHMT49* in Combi199), compared with one parent. It is worth noting that this expression trend is not consistent in all materials. For example, *TaHMT149* and *TaHMT152* in Combi7 has low parental expression. This seems to be a manifestation of the complexity of heterosis. Interestingly, another DEG, *TaHMT39*, showed a low-parent dominant expression pattern in the transgressive materials (HP) and high-parent dominant expression patterns in the low-parent materials (LP) (Fig. 8A).

The high-expression genes in spikes showed multiple expression patterns in all combinations. However, no consistent rule indicated that we could not obtain a more consistent conclusion. *TaHMT71* in the M5 combination, *TaHMT118* in the M6 combination, and *TaHMT162* in the M1 combination had negative correlation expression patterns. In HP, the expression in F_1_ was up-regulated by about 6 times, while in LP, the expression was down regulated by over 25 times at most (Fig. 8B). This means that in the HP materials, the genes presented a trend of low expression and a trend of high expression in the LP material. The expression patterns of *TaHMT83*, *TaHMT84*, and *TaHMT148* were positively correlated in the M4 combination, and their expression trends were consistent with the TSW, showing an over-dominant expression pattern (Fig. 8C). Conversely, *TaHMT8*6, *TaHMT89*, and *TaHMT93* showed under-dominant patterns in some combinations (Fig. 8D).

**Fig. 9:**
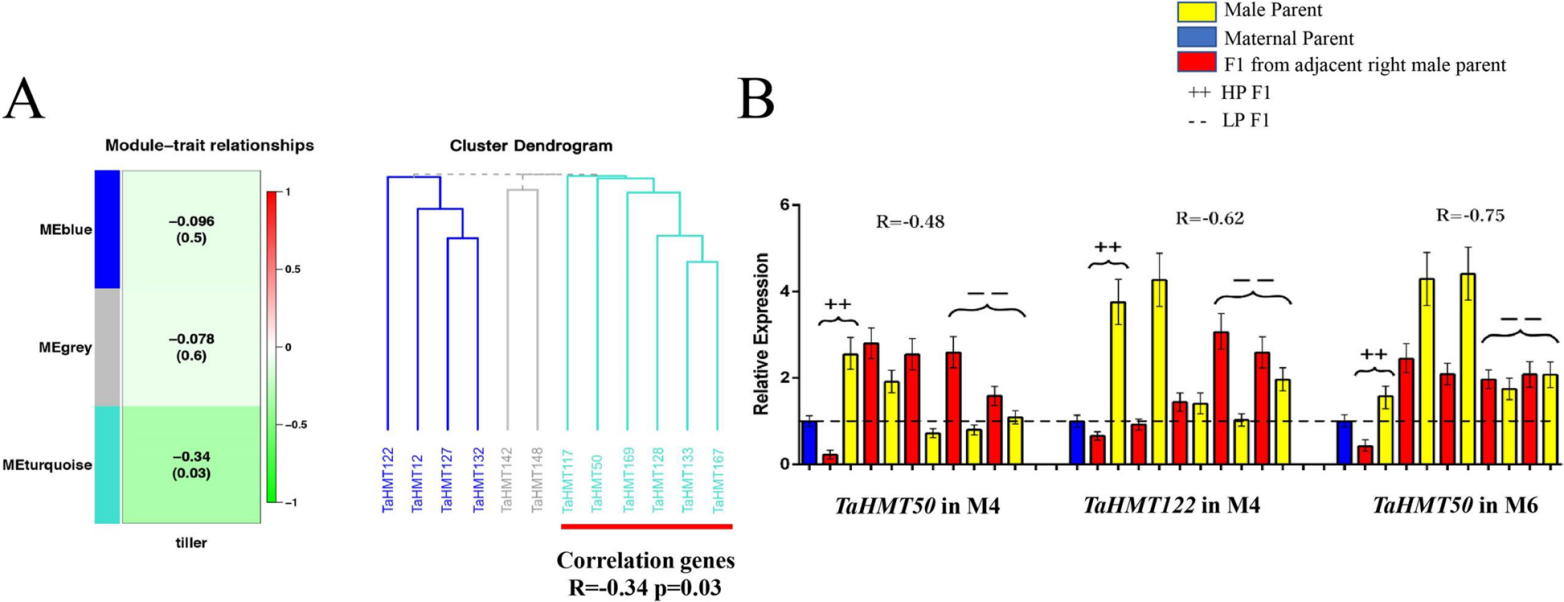
Expression analysis 12 *TaHMT* genes in tillers. **A** WGCNA analysis of 12 genes in tillers. **B** The expression of *TaHMT50* and *TaHMT122* in specific combinations showed a strong negative correlation with effective tiller number. The relative expression of the female parent of each combination was relative 1, which is shown in the blue-colored and marked as a dotted line. Red represented the relative expression of F_1_, and the yellow adjacent to the right represents the relative expression of its male paternity. +  + marks applicability for all HP combinations, and – indicates LP combinations. R represents the Pearson correlation coefficient. Values are means ± SD (*n* = 3)

For the analysis of the expression of the 12 genes in the tillers: as a whole, the expression of the 12 genes had a negative correlation with the number of tillers, but the trend was nevertheless not very significant. No significant correlation was seen between the first two modules and the number of tillers. Therefore, the gene may have no relation to the number of tillers. The expression of the six genes in the third modules (green part) was negatively correlated with the number of tillers (*R* = -0.34, *p* = 0.03). From the analysis of the expression of each combination, the *TaHMT* genes mainly showed a negative correlation (Fig. 9A). That is, the expression of F_1_ was significantly down-regulated in HP and significantly up-regulated in LP. For example, *TaHMT50* was down-regulated by 3–5 times in HP in M6 and M4, and up-regulated by 3 times in LP in M4. In particular, the expression of *TaHMT122* in maternal M4 and *TaHMT50* in M6 had a strong negative correlation (*R* > -0.6, *p* < 0.05) with the tiller number (Fig. 9B).

### The abundance of lncRNAs were associated with histone methylation

**Fig. 10:**
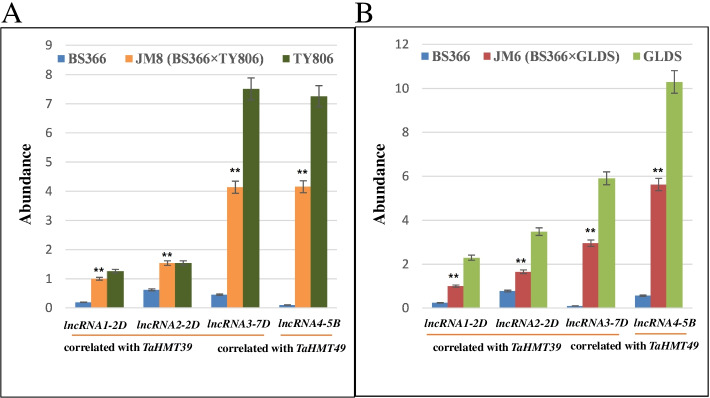
Abundance of four *lncRNA*s in JM8 combination (**A**) and JM6 combinations (**B**). The relative expression levels were significantly those that which met the |fold change|> 2 and *p* < 0.01 in spikes. **indicates a significant difference from the one parent at *p* < 0.01 according to Student’s *t*-test

Studies have shown that the lncRNAs are important regulation factors involved in the methylation of histone [41, 42]. So far, a large number of lncRNAs have been found and studied in *Arabidopsis*, rice, and maize, which are obviously related to the development and reproduction of panicles [43, 44]. We obtained four lncRNAs using the JM6 and JM8 lncRNA sequencing databases. Among the four DEGs, *TaHMT39* and *TaHMT49* showed a strong correlation with these lncRNAs (Fig. 10, Table 4). The expression of these four lncRNAs in the two-line hybrid wheat combinations of JM6(BS366 × GLDS) and JM8(BS366 × TY806) were higher than those in female parents, but lower than that of male parents, showing very similar expression patterns with their possible target genes.

**Table 4:** Correlation analysis of *TaHMT* genes and *lncRNA*s

mRNA	lncRNA (Genome Version: TGACv1)	R	*P*-value	mode
*TaHMT39*	*TRIAE_CS42_2DS_TGACv1_177271_AA0571600 lncRNA1*^a^	0.9504	1.73E-08	cis
	*TRIAE_CS42_2DS_TGACv1_178127_AA0591260 lncRNA2*	0.9541	1.02E-08	cis
	*TRIAE_CS42_7DS_TGACv1_622339_AA2037900 lncRNA3*	0.9639	1.93E-09	trans
*TaHMT49*	*TRIAE_CS42_5BL_TGACv1_404911_AA1314590 LncRNA4*	0.9517	1.44E-08	trans

^a^ Numbered in the order of the tables, R and mode of action are detailed in Materials and Methods

### Three *TaCCA1* genes were regulated by histone methylation

**Fig. 11:**
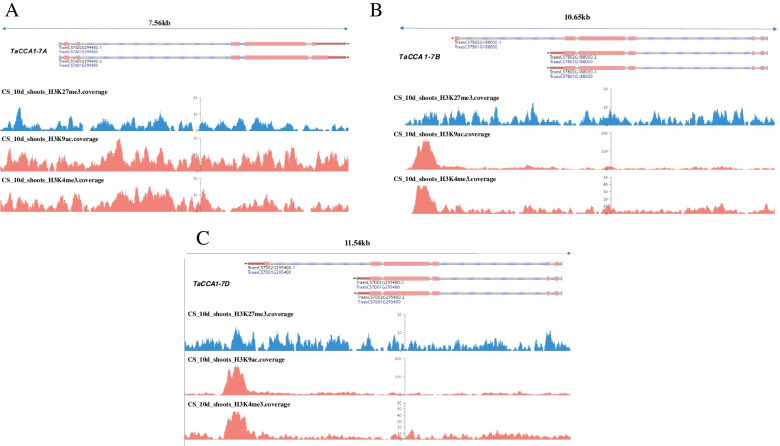
Prediction of histone modification in gene body region and promoter region of three TaCCA1 genes of *TaCCA1-7A* (**A**), *TaCCA1-7B* (**B**) and *TaCCA1-7D* (**C**). From top to bottom of the figure, they are length of the regions, different transcripts of the genes and the abundance of different histone modifications. The results showed that the histone modified region was mainly enriched in the promoter region

**Fig. 12:**
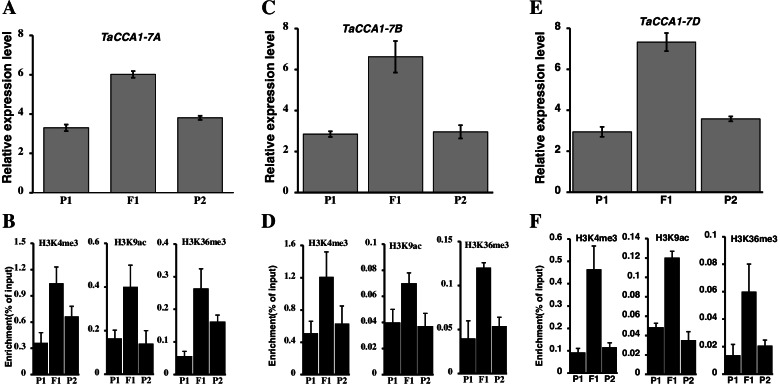
ChIP-qPCR analysis of three *TaCCA1* genes on JM8 combinations. Expression level of *TaCCA1-7A* (**A-B**), *TaCCA1-7B* (**C-D**) and *TaCCA1-7D* (**E–F**) in F_1_ and its parents as well as H3K4me3/H3K9ac/H3K36me3 histone modification analysis of 5kb promoter region of the genes

The growth heterosis of *Arabidopsis* is related to the change of histone modification state in the promoter region of *CCA1* and *LHY*. The apparent regulation of H3K9ac and H3K4me2 reduces the expression of *CCA1* and *LHY*, while the expression of downstream genes controlling photosynthesis and starch metabolism increases accordingly, which improves the photosynthetic utilization efficiency, starch accumulation and growth heterosis of hybrid F_1_ [20]. We predicted the methylation level of *TaCCA1* promoter regions and gene body regions at seedling stage (Fig. 11). It was found that the three homologous *TaCCA1s* were rich in a large number of histone modifications such as H3K4me3 and H3K9ac in the promoter regions. The expression of *TaCCA1* in JM8 was significantly higher than that in its parents, and the results of ChIP-qPCR showed that histone modification level in the 5 kb promoter regions were also significantly higher than that in its parents (Fig. 12).

## Discussion

### Characteristics comparison of *HMT* genes in wheat and other diploid species

Allopolyploidization is the key event in the formation of common wheat as one of the main reasons for environmental adaptability [45]. Histone methylation modifying genes are widely seen in animals and plants. The increased multi-function of the SET gene evolution may ensure plants to be more adaptive to the complex natural environment [46]. In this study, the number of *TaHMT* genes identified was about three times than other five diploids and shows that the gene family has expanded. We found that 92.76% of the HKMT genes were triplet genes which is a typical characteristic of hexaploid wheat. Comparing the number of genes in each subfamily in wheat, *Arabidopsis,* and rice, the main reason is the expansion of gene number in class I E(z)-like and class V Su(var) 3–9-like, while PRMTs is relatively conservative. our study is consistent with previous studies which have shown that the SUV subfamily shows the rapid evolution [36]. We also found that ASH-like also has a high evolution speed. The ratio of this subfamily in wheat to its amount in rice and *Arabidopsis* was significantly higher than the average.

Wheat has more orthologous genes and paralogous genes than other diploid species, which is a typical characteristics of a wheat heteropolyploid. According to the homology analysis, the ortholog group of wheat achieved a higher average size of 2.32, and therefore, every *TaHMT* gene had an average of 1.32 paralogs. This number is higher than other species, which supports the previous results in wheat [47]. In wheat, 67.11% of SET gene members belong to Suv, Ash, Trx and E(z) families, and most of them have colinearity support, showing that the diversity of SET gene is mainly caused by genome duplication. We also observed that, in addition to the SET protein domain, HKMT has evolved more new protein structures such as pre-SET, SRA, AWS, WIYLD, and SANT, among which SRA and WIYLD are structures plant-specific [48]. In fact, the presence of more auxiliary proteins such as pre-SET and post-SET may make the function of the SET protein more stable. Histone methylation can activate or repress gene expression according to different modified types. The modification types of each subfamily of HMT protein have been widely studied. For example, the activated sites (H3K36 and H3K4) include subfamilies of class II, class III, class IV and class VI, while the exercise inhibited sites (H3K9 and H3K27) contains the subfamilies of class I and class V. Unlike HKMTs, PRMTs are relatively conservative. The role of PRMTs in plants is far less clear than that of HKMTs [49]. Our results show that PRMTs are mainly located in the cytoplasm, unlike the nucleus location of HKMTs, and their physical and chemical properties tend to be consistent. According to the previous studies, PRMTs is mainly divided into three types of arginine methylation: ω-NG monthyllargine (MMA), ω-NG, NG-asymmetric dimethylargine (aDMA), and ω-NG, N’G-symmetric dimethylargine (sDMA) [50]. One type of PRMT can often catalyze two methylation modes. The same arginine measurement plays a major role in general regulation because of the capability of the PRMTs to deposit key activation functions (H4R3me2a, H3R2me2s, H3R17me2a and H3R26me2a) or repressive functions (H3R2me2a, H3R8me2a, H3R8me2a and H3R8me2s) [51]. The phylogenetic tree shows that wheat contains four types of PRMTs, while their catalytic sites and functions need further study.

### Histone methylation may be involved in regulating heterosis in two-line hybrid wheat

The utilization of heterosis is the main mean of improving crop yield. Heterosis utilization can be accelerated by using male sterile lines. In wheat, TGMS lines (such as BS366) are the main means of heterosis utilization. Many excellent two-line hybrid wheat varieties with good yield performance were developed by TGMS as maternal materials [52]. The male sterility of TGMS can be inherited and has some epigenetic characteristics. Through the analysis of tissue-specific expression of TaHMTs using Chinses Spring (CS) wheat expression data in this study we found that spike was the concentrated expression tissue, and its expression was concentrated in the vegetative and reproductive period. This is consistent with the previous work, which showed that HMTs were closely related to the development of flower organs and flowering [53]. The transcriptome data of two-line hybrid wheat is to a certain degree consistent with CS expression data. They share a number of genes that are highly expressed in both spike and shoot. The expression analysis of hybrid combinations with different yield performance showed that the correlation between the relative expression of 31 genes and their yield traits is relatively low, which may be due to the lower gene expression levels in the experimental context, the complexity of quantitative traits controlled by multiple genes, and the uncertainty of apparent modification [54, 55]. Among the known modes of microRNAs regulating target genes, the two main ways are miRNAs direct target mRNAs cleavage and translational repression [56]. Of the fifteen target sites we obtained, thirteen of them were predicted to act in a cleavage mode indicating a main way for miRNA to regulate *HMT* genes in wheat. In rice, some miRNAs have been identified to be involved in controlling rice grain size and panicle branching by targeting severe SPL genes, including *OsSPL14* and *OsSPL16* [57–59]. In our study, the expression of *TaHMT162* in M1 combination, *TaHMT71* in M5 combination showed a significant negative correlation with TSW, while the gene expression of *TaHMT50* in M1 and M6 combination also showed a significant negative correlation with the number of effective tillers. In the regulation of miRNAs, there are cleavage-regulation modes of *tae-miR1122b-3p*-*TaHMT162* and *tae-miR9677a*-*TaHMT50/71*. We speculated that the histone methylation catalyzed by these three *HMT* genes might negatively regulate the expression of yield-related genes, and the control of miRNA reduces their mRNAs, resulting in low expression in HP but high expression in LP. Whether their regulatory roles are similar to those in rice need further more experimental evidences. It was also found that four DEGs had significant expression differences in JM8 hybrid combinations, which were also reflected in other two-line hybrid wheat combinations. Among the four DEGs, *TaHMT39* and *TaHMT49* exhibited a strong correlation with the expression of four lncRNAs in the two-line hybrid wheat combinations of JM6 and JM8 with both cis-regulation mode and trans-regulation mode. This result also further indicated that these DEGs may function positively in the process of heterosis regulated by these lncRNAs. Heterosis is a complex biological phenomenon. The interaction networks provide a theoretical foundation for further study on the methylation of candidate genes and the influence of non-coding RNA on plant growth and development. The cooperative relationship between miRNA-lncRNA-HMT gene will be a promising study for the analysis of heterosis in wheat.

In *Arabidopsis* hybrids and allopolyploids, increased photosynthetic and metabolic activities are linked to altered expression of circadian clock regulators, including *CIRCADIAN CLOCK ASSOCIATED1 (CCA1)* [20]. The higher levels of carbon fixation and starch accumulation in the maize hybrids are also associated with altered temporal gene expression and overexpressing ZmCCA1b disrupts circadian rhythms and biomass heterosis [60]. However, this may have a different mechanism from that of dicotyledonous plants. In *Arabidopsis*, *CCA1* modified by H3K9ac and H3K4me2 reduce the expression level, which improves the expression of downstream genes regulating photosynthesis and starch metabolism. Our findings indicated that the expression of three *TaCCA1* genes in hybrid with heterosis were higher than that in parents, and the methylation modifications of H3K4me3/H3K9ac and H4K36me3 activated gene expression in promoter regions were also significantly enrichment. This mechanism is similar to that of monocotyledonous maize, which may be an important way for histone methylation to participate in the heterosis of hybrid wheat. Although the relationship between gene expression and histone modification is consistent with previous knowledge [21, 22], the differences of expression patterns of *CCA1* among species and how to accurately control the expression of *CCA1* by the histone modifications to achieve heterosis are worthy of in-depth research. At the same time, some important genes associated with heterosis for the location of histone methylation modifications are also noteworthy in the future.

## Conclusion

In this study, two types of histone methylation genes, histone lysine methylation gene and histone arginine methylation gene, a total of 175 members were comprehensively identified in wheat as well as its relative species, and the functions of the genes in terms of structure, evolution and expression regulation were deeply explored. Through transcriptome sequencing and analysis of expression in different yield performance hybrid combinations, we found some potential DEGs that may be involved in the construction of yield-heterosis. Furthermore, the identification and expression analysis of lncRNAs with strong correlation to the DEGs were conducted to elucidate their roles for yield-heterosis. Furthermore, the high expression and high histone modification levels of *CCA1* in hybrid F_1_ indicated that histone methylation played important potential roles in regulating yield traits heterosis. Our results accumulated the original data for the cloning of functional genes in the future, and laid a certain data foundation for in-depth research on the growth and development of wheat and the improvement of yield.

## Materials and methods

### Identification of *HMT* genes in wheat and its relatives

To identify the putative *HMT* genes, the wheat genome sequence, gene annotation files, and protein sequences were downloaded from the ENSEMBL database (http://plants.ensembl.org/Triticum_aestivum/Info/Index: version: release-46, as well as http://oct2017-plants.ensembl.org/index.html: TGACv1). The genome and protein data for *Sorghum bicolor*, *Hordeum vulgare*, and *Brachypodium distachyon* were downloaded simultaneously. The Hidden Markov Model (HMM version 33.0) was conducted using the profiles of the SET domain (Pfam accession No: PF00856) as queries with the hmmsearch program of all annotated proteins of these species (E < 1e^−10^). BLAT was used to compare the protein sequences of each species with the Pfam sequence of the gene family (the following analysis software used the default parameters unless otherwise specified). The modified DOT1L (Pfam accession No: PF08123), which participates in H3K79 methylation, was the only lysine methyltransferase that has so far been found without the SET-conserved domain [61]. We did not find genes containing the DOT1L conserved domain in wheat.

PRMT5 is the most important type II protein arginine methyltransferase. It catalyzes the formation of symmetrical arginine demethylation [62]. Consequently, the Hidden Markov Model was also conducted using the HMM profiles of PRMT5 domain (Pfam accession No: PF05185). The nine reported *Arabidopsis* PRMTs and the eight reported rice PRMTs were used to conduct a BLASTP program (Version: blast-2.10) with a wheat protein database. The standard of the BLASTP is as follows: (1) e-value < 1e^−10^; (2) bit-score > 100 and global alignment of identity > 50% overlap, that is, the length of the matching length must exceed 50% of the longer sequence. All of the gene loci were numbered and mapped onto corresponding chromosomes, and the non-chromosome sequences scattered across the genome were merged into unfound-chromosomes (U). The name of each gene begins with Ta, an abbreviation for *Triticum aestivum* L., and the gene loci were re-numbered based on the order of A/B/D subgroups.

### Analysis of the characteristics of *TaHMT* genes

The CD searches (https://www.ncbi.nlm.nih.gov/Structure/cdd/wrpsb.cgi) and ORF finder (https://www.ncbi.nlm.nih.gov/orffinder) functions of NCBI were used to find the location of each gene and the location of ORF reading frame, and ProtParam (http://web.expasy.org/protparam/) was used to predict the molecular weight, isoelectric point, and the instability index of all HMT protein [63, 64]. It is considered to be an unstable protein when the instability index is greater than 40. Subcellular localization was analyzed using cell-ploc2.0 (http://www.csbio.sjtu.edu.cn/bioinf/Cell-PLoc-2/). All genes’ exon–intron structures were depicted by the online tool GSDS (http://gsds.cbi.pku.edu.cn) [65]. Protein organization sketch maps were drawn using Dog2.0 (http://dog.biocuckoo.org/) [66]. The 2 kb sequence before the CDS of each HMT gene was extracted as the promoter region and submitted to PlantCARE database (http://bioinformatics.psb.ugent.be/webtools/plantcare/html/) for cis-regulatory response elements prediction [67], and the results were visualized by TBtools [68]. The miRNAs annotated in wheat and their targets to *HMT*s as well as 2 kb promoter sequences were predicted using the psRNATarget server (https://plantgrn.noble.org/psRNATarget/) with default parameters; the mismatch expectation was 0–3 [69]. The Cytoscape tool was used to visualize the interaction networks of miRNAs and *HMT* targets [70]. The histone modification predicted data of *TaCCA1-7A*, *TaCCA1-7B* and *TaCCA1-7D* are from Chinese Spring Wheat at 10-day seedling stage on WheatOmics database [71].

### Evolutionary analysis of wheat *TaHMT* gene family

Multiple sequence alignment and the phylogenetic analysis of the *HMT* gene family were performed using MUSCLE. InParanoid 4.1 was adopted to analyze the homologous protein (OG, orthologous groups) for every two species, and Multi-Paranoid was used for analyzing multiple species OG [72]. All *HMT* genes were mapped to their respective locus in the wheat genome in a circular diagram using shinyCircos [73]. The HMT proteins for wheat, rice, and *Arabidopsis* were introduced into MEGA-X software to construct phylogenetic trees using Maximum likelihood method [74]. In order to obtain more reliable evolutionary analysis, we constructed the phylogenetic trees of HKMT and PRMT of six species at the same time in Figure S5.

### Expression analysis of *TaHMT* gene family

From the expVIP-Wheat Browser (http://wheat-expression.com/ version: TGACv1), the expression data of root, shoot, leaf, spike, and grain of all *TaHMT*s in seedling, vegetative, and reproductive growth stages were downloaded. The heatmap for gene expression patterns was generated with the online toolshttps://www.chiplot.online/#Heatmap refer to the instructions on the website. The 28 *TaHMT* genes with the highest expression of shoots at the seedling stage and spikes at the reproductive growth stage were screened. In screening for differentially expressed genes (DEGs) from the parents, the two-line hybrid wheat JM8 (Jing Mai 8), cultivated by Beijing Hybrid Wheat Engineering Technology Research Center, was used for RNA sequencing. The transcriptome data can be found in the National Center for Biotechnology Information (NCBI) Sequence Read Archive (SRA) under accession number PRJNA398700 (https://www.ncbi.nlm.nih.gov/bioproject/?term=PRJNA398700) from JM8 and parental ones (BS366 × TY806) [75]. The DEGs in each comparison from among all *TaHMT* genes were identified by EdgeR, using a significance threshold of *P* < 0.05 and a fold change ≥ 2. The first 30 *TaHMT* genes with the highest expression were also screened. To clarify the relationship between the two groups’ data, we used a Venn Diagram (http://bioinformatics.psb.ugent.be/webtools/Venn/) to analyze all of the highest-expression genes.

### Plant materials and treatments

All plant materials used in this article were provided by Beijing Engineering Research Center for Hybrid Wheat. Two-line hybrid wheat JM6, JM8 and other materials have been approved and widely planted in China. A total of 201 two-line hybrid wheat hybridized combinations were prepared with six female parents (M1, M2, M3, M4, M5, and M6) and 34 male parents. These materials, grown at the experimental farm in Beijing Haidian District (China, N39°54′, E116°18′), were regularly watered and fertilized. Each material was planted in three rows as three biological replicates. The corresponding next generations were numbered Combi1 to Combi201 (also 1 to 201 in figures), and the male parents were identified with a prefixed P to the label, such as in P5 or P13. Tillers from root base to stem about 0.5 cm in length were obtained, which contained the apical meristem. As the wheat grew into the anther separation stage, we sampled the spikes with the awn removed. All plant materials used for the above studies were frozen immediately in liquid nitrogen and stored at -80 °C for RNA extraction.

### Collection of yield phenotypes of multiple two-line hybrids

When the wheat was fully matured, all of the samples were collected according to the names of the materials. The number of tillers and spikes with well-grown individuals was investigated. We selected five wheat plants randomly and counted tillers with mature grains. The mature grains were dried and threshed, and 1000 seeds were weighed three times. The yield character data obtained above were recorded, and finally, the dominant differences in F_1_ and parents were evaluated by Student’s *t*-test, *p* < 0.05. The phenotypes and data statistics were listed in Supplementary Figure S4 and Table S6.

### Correlation analysis

The expression of the genes was identified as over-dominance or under-dominance if the expression level of a gene in F_1_ was significantly higher or lower than, respectively, the expression level in both parents. The high-parent dominant genes and low-parent dominant genes were defined such that the expression level in F_1_ genotype was significantly different from that in only one parent. Phenotypic data were processed in a similar way to the genotype expression. The materials were divided into high-parent material (HP), middle-parent material (MP), and low-parent material (LP). The yield data of five maternal parents in the hybrid combinations were homogenized. Pearson analysis was conducted to calculate the correlation between yield-character data and gene-expression data after homogenization using SPSS27.0 software. The Pearson correlation coefficient (R) was calculated, and the *p*-value was considered statistically significant when it was below 0.05. To identify discrete groups of co-expressed genes showing their relationship to multiple traits, we applied TKW/Tillers-*HMT* weighted gene co-expression network analysis (WGCNA) to integrate expression differences into a higher order. The network was built using 42 wheat samples. Eigengenes (the normalized linear combination of genes with the largest variance in a population) carry the representative gene expression pattern for each module. The association between co-expressed gene modules and phenotypic traits was further assessed using Pearson correlations. Based on our sequencing data of lncRNA of Two-line Hybrid Wheat JM6 and JM8 combinations, we compared the lncRNA with known mRNA and used the results of Cufflinks to screen candidate lncRNA. According to the structural characteristics and noncoding functional characteristics of lncRNA, the assembling results based on StringTie software are screened. LncRNA functions mainly through “cis” or “trans” acting on protein coding target genes. For the prediction of cis target genes, we screened the (upstream and downstream 100 k) protein coding genes near the lncRNA as their target genes. Subsequently, the main functions of lncRNA were predicted by target gene function enrichment analysis. For the prediction of “trans” target genes, we used Pearson correlation coefficient to analyze the correlation between lncRNA and target genes expression among samples, and took the protein coding genes with high correlation for function enrichment analysis to predict the main functions of lncRNA. The Pearson correlation coefficient (R) was calculated using SPSS 27.0 and a multiple linear regression analysis was applied in the bivariate analysis. A *t*-test was used for comparison among groups.

### qRT-PCR and ChIP-qPCR

For the expression analysis of *HMT* genes, total RNA was extracted from plant tissues using TRIzol reagent (Ambion, USA) according to the manufacturer’s instructions. First-strand cDNA synthesis was performed using a TaKaRa PrimeScript™ RT Reagent Kit with gDNA Eraser (TaKaRa, Dalian, China). qRT-PCR analysis was conducted using an CFX96 Touch™ Real-Time PCR Detection System (Bio-Rad Laboratories, Hercules, CA, USA) with Takara SYBR® Premix Ex Taq™ (Tli RNaseH Plus). The primers for all genes were designed using the Primer3plus online tools (http://www.primer3plus.com/cgi-bin/dev/primer3plus.cgi). Each reaction was performed in triplicate in a reaction volume of 10 µL. Expression levels of genes in samples were normalized using endogenous wheat *18 s* gene; the relative expression levels were calculated using the 2^−ΔΔCt^ method [76]. Chromatin Immunoprecipitation (ChIP) Assay Followed by qPCR (ChIP-qPCR). Chromatin immunoprecipitation assays were performed according to a previously described method [77]. Briefly, 2 g of samples were washed twice in cold PBS buffer, and proteins were cross-linked to DNA by incubating the samples with formaldehyde at a final concentration of 1% on a shaking device for 10 min at 4 °C. Samples were then lysed, and chromatin was precipitated on ice. Chromatin was then sonicated to yield soluble sheared chromatin (200–500 bp). One part of the soluble chromatin was saved at -20 °C for input DNA, and the remainder was used for immunoprecipitation with antibodies for H3K9Ac (CST, 9649), H3K4Me3 (CST, 9751), H3K36Me3 (CST, 4909), and normal rabbit IgG (CST, 2729). Immunoprecipitated DNA was amplified by PCR using their specific primers. PCR reactions were set up and run using the ChamQ SYBR Color qPCR Master Mix. The enrichment values were normalized to the input sample. The primers used are shown in Supplementary Table S7.

## Supplementary Information

**Additional file 1:** **Figure S1.** (A) and (B): Distribution of two sub-gene families on wheat chromosome. Distribution of two gene families on wheat chromosome. All wheat chromosomes have HKMT gene distribution, which are relatively uniform. However, chromosome 2 and chromosome 3 also tend to cluster. Compared with the HKMT genes, the number of PRMT genes is relatively small, and the distribution of PRMT genes in the whole wheat genome is uneven. In addition, compared with previous research results, most HMT genes have been located in a clear range, and there is only one HKMT gene on the U chromosome, may also indicate that the genome quality of wheat has been greatly improved. **Figure S2.** Analysis of cis-elements of promoters of TaHMT genes. The 2kb sequence before the CDS of each HMT gene was extracted as the promoter region and submitted to PlantCARE database for cis-regulatory response elements prediction, and the results were visualized by TBtools. **Figure S3.** Expression analysis all selected TaHMT genes. (A) Expression analysis of 19 genes in spike. (B) Expression analysis of 12 genes in tiller. The relative expression of the female parent of each combination was relative 1, and the expression values for each parent are placed in front of each combination. The horizontal column is the gene name and the vertical column is the name of the samples. **Figure S4.** The phenotype as well as sampling times at different periods of wheat development. (A) Different periods of wheat spike development and sampling were conducted on this basis at anther separation stage. The length of the red ruler represents 1mm. (B) Tillers from root base to stem about 0.5 cm in length were sampled which contains the apical meristem at about four-leaf stage; P: male parent, M: maternal parent. (C) Phenotypic trait combinations for different tillers at harvest time, from left to right, they were: high parent (HP), middle parent (MP) and low parent (LP). In the picture, the three plants were photographed as a whole. **Figure S5.** The phylogenetic trees of HKMT (A) and PRMT (B) proteins from wheat, rice, Arabidopsis, Sorghum bicolor, Hordeum vulgare, and Brachypodium distachyon were constructed by MEGA-X using Maximum likelihood method. The HKMT proteins were divided into seven subfamilies and PRMT were divided into four subfamilies based on the previous methods. The members of wheat were marked with red circles in Fig. S5B. **Figure S6.** Interaction networks of miRNAs and HMT targets. miRNAs were marked as a blue node and HMT targets were marked as a yellow node. The size of the node represents the number of action sites. The black solid line represents that the mismatch expectation is 3.0, the red thick solid line represents that the mismatch expectation is 1.5, and the green thick solid line represents that the mismatch expectation is 2.5. 

**Additional file 2:** **Table S1.** Basic characteristic features of 175 HMT genes identified in wheat.

**Additional file 3:** **Table S2.** Location and Homoeologous of HMT genes in wheat.

**Additional file 4:** **Table S3.** Distribution of HMT family members in subgroups in wheat, Arabidopsis and rice.

**Additional file 5:** **Table S4.** Targeting analysis of miRNAs and HMT genes in wheat.

**Additional file 6:** **Table S5.** mRNA abundance of top 30 highly expressed genes.

**Additional file 7:** **Table S6.** Phenotypic statistics. **Table S6-1.**The number of the tiller. **Table S6-2.** Thousand seed weight (TSW) (g).

**Additional file 8:** **Table S7.** Primers used for qRT-PCR and ChIP-qPCR.

## Data Availability

The genome databases of wheat and other plants used are derived from http://plants.ensembl.org/index.html. The expression data of wheat comes from http://wheat-expression.com/. The transcriptome data of Two-line Hybrid Wheat (JM8) were derived from https://www.ncbi.nlm.nih.gov/bioproject/?term=PRJNA398700. We declare that the datasets required to reproduce the results of this article are included in the article and additional files available.

## Abbreviations

TaHMT: Wheat Histone Methyltransferase
H3K4me3: Histone H3 lysine 4 tri-methylation
HKMT: Histone lysine methyltransferase
PRMT: Protein arginine methyltransferase
CS wheat: Chinese Spring wheat
TSW: Thousand seeds weight
RNA-seq: RNA sequencing

## References

[1] Brenchley R, Spannagl M, Pfeifer M, Barker GL, D'Amore R, Allen AM, McKenzie N, Kramer M, Kerhornou A, Bolser D (2012). Analysis of the bread wheat genome using whole-genome shotgun sequencing. Nature.

[2] Shewry PR (2009). Wheat. J Exp Bot.

[3] Avni R, Nave M, Barad O, Baruch K, Twardziok SO, Gundlach H, Hale I, Mascher M, Spannagl M, Wiebe K (2017). Wild emmer genome architecture and diversity elucidate wheat evolution and domestication. Science.

[4] He F, Pasam R, Shi F, Kant S, Keeble-Gagnere G, Kay P, Forrest K, Fritz A, Hucl P, Wiebe K (2019). Exome sequencing highlights the role of wild-relative introgression in shaping the adaptive landscape of the wheat genome. Nat Genet.

[5] International Wheat Genome Sequencing C, investigators IRp (2018). Shifting the limits in wheat research and breeding using a fully annotated reference genome. Science.

[6] Ramirez-Gonzalez RH, Borrill P, Lang D, Harrington SA, Brinton J, Venturini L, Davey M, Jacobs J, van Ex F, Pasha A (2018). The transcriptional landscape of polyploid wheat. Science.

[7] Walkowiak S, Gao L, Monat C, Haberer G, Kassa MT, Brinton J, Ramirez-Gonzalez RH, Kolodziej MC, Delorean E, Thambugala D (2020). Multiple wheat genomes reveal global variation in modern breeding. Nature.

[8] Bird A (2007). Perceptions of epigenetics. Nature.

[9] Egger G, Liang G, Aparicio A, Jones PA (2004). Epigenetics in human disease and prospects for epigenetic therapy. Nature.

[10] Holliday R (1987). DNA methylation and epigenetic defects in carcinogenesis. Mutat Res.

[11] Liu C, Lu F, Cui X, Cao X (2010). Histone methylation in higher plants. Annu Rev Plant Biol.

[12] Ay N, Janack B, Humbeck K (2014). Epigenetic control of plant senescence and linked processes. J Exp Bot.

[13] Huang Y, Mo Y, Chen P, Yuan X, Meng F, Zhu S, Liu Z (2016). Identification of SET domain-containing proteins in gossypium raimondii and their response to high temperature stress. Sci Rep.

[14] Niu L, Lu F, Zhao T, Liu C, Cao X (2012). The enzymatic activity of Arabidopsis protein arginine methyltransferase 10 is essential for flowering time regulation. Protein Cell.

[15] Thorstensen T, Grini PE, Aalen RB (2011). SET domain proteins in plant development. Biochim Biophys Acta.

[16] Yi X, Jiang XJ, Li XY, Jiang DS (2015). Histone methyltransferases: novel targets for tumor and developmental defects. Am J Transl Res.

[17] Wang X, Zhang Y, Ma Q, Zhang Z, Xue Y, Bao S, Chong K (2007). SKB1-mediated symmetric dimethylation of histone H4R3 controls flowering time in Arabidopsis. EMBO J.

[18] Comings DE, MacMurray JP (2000). Molecular heterosis: a review. Mol Genet Metab.

[19] He G, Chen B, Wang X, Li X, Li J, He H, Yang M, Lu L, Qi Y, Wang X (2013). Conservation and divergence of transcriptomic and epigenomic variation in maize hybrids. Genome Biol.

[20] Ni Z, Kim ED, Ha M, Lackey E, Liu J, Zhang Y, Sun Q, Chen ZJ (2009). Altered circadian rhythms regulate growth vigour in hybrids and allopolyploids. Nature.

[21] Jenuwein T, Allis CD (2001). Translating the histone code. Science.

[22] Li B, Carey M, Workman JL (2007). The role of chromatin during transcription. Cell.

[23] Moghaddam AM, Roudier F, Seifert M, Berard C, Magniette ML, Ashtiyani RK, Houben A, Colot V, Mette MF (2011). Additive inheritance of histone modifications in Arabidopsis thaliana intra-specific hybrids. Plant J.

[24] Kim DH, Sung S (2012). Environmentally coordinated epigenetic silencing of FLC by protein and long noncoding RNA components. Curr Opin Plant Biol.

[25] Tian Y, Zheng H, Zhang F, Wang S, Ji X, Xu C, He Y, Ding Y (2019). PRC2 recruitment and H3K27me3 deposition at FLC require FCA binding of COOLAIR. Sci Adv.

[26] Heo JB, Sung S (2011). Vernalization-mediated epigenetic silencing by a long intronic noncoding RNA. Science.

[27] Swiezewski S, Liu F, Magusin A, Dean C (2009). Cold-induced silencing by long antisense transcripts of an Arabidopsis Polycomb target. Nature.

[28] Rubio-Somoza I, Weigel D (2011). MicroRNA networks and developmental plasticity in plants. Trends Plant Sci.

[29] Tang J, Chu C (2017). MicroRNAs in crop improvement: fine-tuners for complex traits. Nat Plants.

[30] Ahmad A, Dong Y, Cao X (2011). Characterization of the PRMT gene family in rice reveals conservation of arginine methylation. PLoS ONE.

[31] Aiese Cigliano R, Sanseverino W, Cremona G, Ercolano MR, Conicella C, Consiglio FM (2013). Genome-wide analysis of histone modifiers in tomato: gaining an insight into their developmental roles. BMC Genomics.

[32] Batra R, Gautam T, Pal S, Chaturvedi D, Jan RI, Balyan HS, Gupta PK (2020). Identification and characterization of SET domain family genes in bread wheat (Triticum aestivum L.). Sci Rep.

[33] Fan S, Wang J, Lei C, Gao C, Yang Y, Li Y, An N, Zhang D, Han M (2018). Identification and characterization of histone modification gene family reveal their critical responses to flower induction in apple. Bmc Plant Biol.

[34] Qian Y, Xi Y, Cheng B, Zhu S, Kan X (2014). Identification and characterization of the SET domain gene family in maize. Mol Biol Rep.

[35] Xu J, Xu H, Liu Y, Wang X, Xu Q, Deng X (2015). Genome-wide identification of sweet orange (Citrus sinensis) histone modification gene families and their expression analysis during the fruit development and fruit-blue mold infection process. Front Plant Sci.

[36] Zhang LS, Ma CR, Ji Q, Wang YF (2009). Genome-wide identification, classification and expression analyses of SET domain gene family in Arabidopsis and rice. Yi Chuan.

[37] International Brachypodium I (2010). Genome sequencing and analysis of the model grass Brachypodium distachyon. Nature.

[38] Schilling S, Kennedy A, Pan S, Jermiin LS, Melzer R (2020). Genome-wide analysis of MIKC-type MADS-box genes in wheat: pervasive duplications, functional conservation and putative neofunctionalization. New Phytol.

[39] Ng DW, Wang T, Chandrasekharan MB, Aramayo R, Kertbundit S, Hall TC (2007). Plant SET domain-containing proteins: structure, function and regulation. Biochim Biophys Acta.

[40] Bedford MT (2007). Arginine methylation at a glance. J Cell Sci.

[41] Magistri M, Faghihi MA, St Laurent G, Wahlestedt C (2012). Regulation of chromatin structure by long noncoding RNAs: focus on natural antisense transcripts. Trends Genet.

[42] Tsai MC, Manor O, Wan Y, Mosammaparast N, Wang JK, Lan F, Shi Y, Segal E, Chang HY (2010). Long noncoding RNA as modular scaffold of histone modification complexes. Science.

[43] Li L, Eichten SR, Shimizu R, Petsch K, Yeh CT, Wu W, Chettoor AM, Givan SA, Cole RA, Fowler JE (2018). Correction to: Genome-wide discovery and characterization of maize long non-coding RNAs. Genome Biol.

[44] Zhang YC, Liao JY, Li ZY, Yu Y, Zhang JP, Li QF, Qu LH, Shu WS, Chen YQ (2014). Genome-wide screening and functional analysis identify a large number of long noncoding RNAs involved in the sexual reproduction of rice. Genome Biol.

[45] Feldman M, Levy AA (2012). Genome evolution due to allopolyploidization in wheat. Genetics.

[46] Fawcett JA, Maere S, Van de Peer Y (2009). Plants with double genomes might have had a better chance to survive the cretaceous-tertiary extinction event. Proc Natl Acad Sci U S A.

[47] Li X, Gao S, Tang Y, Li L, Zhang F, Feng B, Fang Z, Ma L, Zhao C (2015). Genome-wide identification and evolutionary analyses of bZIP transcription factors in wheat and its relatives and expression profiles of anther development related TabZIP genes. BMC Genomics.

[48] Freiman RN, Tjian R (2003). Regulating the regulators: lysine modifications make their mark. Cell.

[49] Paik WK, Paik DC, Kim S (2007). Historical review: the field of protein methylation. Trends Biochem Sci.

[50] Blanc RS, Richard S (2017). Arginine methylation: the coming of age. Mol Cell.

[51] Auclair Y, Richard S (2013). The role of arginine methylation in the DNA damage response. DNA Repair (Amst).

[52] Tang Z, Zhang L, Xu C, Yuan S, Zhang F, Zheng Y, Zhao C (2012). Uncovering small RNA-mediated responses to cold stress in a wheat thermosensitive genic male-sterile line by deep sequencing. Plant Physiol.

[53] Schubert D, Primavesi L, Bishopp A, Roberts G, Doonan J, Jenuwein T, Goodrich J (2006). Silencing by plant Polycomb-group genes requires dispersed trimethylation of histone H3 at lysine 27. EMBO J.

[54] Swanson-Wagner RA, Jia Y, DeCook R, Borsuk LA, Nettleton D, Schnable PS (2006). All possible modes of gene action are observed in a global comparison of gene expression in a maize F1 hybrid and its inbred parents. Proc Natl Acad Sci U S A.

[55] Zhang HY, He H, Chen LB, Li L, Liang MZ, Wang XF, Liu XG, He GM, Chen RS, Ma LG (2008). A genome-wide transcription analysis reveals a close correlation of promoter INDEL polymorphism and heterotic gene expression in rice hybrids. Mol Plant.

[56] Song X, Li Y, Cao X, Qi Y (2019). MicroRNAs and their regulatory roles in plant-environment interactions. Annu Rev Plant Biol.

[57] Jiao Y, Wang Y, Xue D, Wang J, Yan M, Liu G, Dong G, Zeng D, Lu Z, Zhu X (2010). Regulation of OsSPL14 by OsmiR156 defines ideal plant architecture in rice. Nat Genet.

[58] Miura K, Ikeda M, Matsubara A, Song XJ, Ito M, Asano K, Matsuoka M, Kitano H, Ashikari M (2010). OsSPL14 promotes panicle branching and higher grain productivity in rice. Nat Genet.

[59] Wang S, Wu K, Yuan Q, Liu X, Liu Z, Lin X, Zeng R, Zhu H, Dong G, Qian Q (2012). Control of grain size, shape and quality by OsSPL16 in rice. Nat Genet.

[60] Ko DK, Rohozinski D, Song Q, Taylor SH, Juenger TE, Harmon FG, Chen ZJ (2016). Temporal shift of circadian-mediated gene expression and carbon fixation contributes to biomass heterosis in maize hybrids. PLoS Genet.

[61] Gao WL, Liu HL (2007). DOT1: a distinct class of histone lysine methyltransferase. Yi Chuan.

[62] Peterson CL, Laniel MA (2004). Histones and histone modifications. Curr Biol.

[63] Marchler-Bauer A, Derbyshire MK, Gonzales NR, Lu S, Chitsaz F, Geer LY, Geer RC, He J, Gwadz M, Hurwitz DI (2015). CDD: NCBI's conserved domain database. Nucleic Acids Res.

[64] Wilkins MR, Gasteiger E, Bairoch A, Sanchez JC, Williams KL, Appel RD, Hochstrasser DF (1999). Protein identification and analysis tools in the ExPASy server. Methods Mol Biol.

[65] Hu B, Jin J, Guo AY, Zhang H, Luo J, Gao G (2015). GSDS 2.0: an upgraded gene feature visualization server. Bioinformatics.

[66] Ren J, Wen L, Gao X, Jin C, Xue Y, Yao X (2009). DOG 1.0: illustrator of protein domain structures. Cell Res.

[67] Lescot M, Dehais P, Thijs G, Marchal K, Moreau Y, Van de Peer Y, Rouze P, Rombauts S (2002). PlantCARE, a database of plant cis-acting regulatory elements and a portal to tools for in silico analysis of promoter sequences. Nucleic Acids Res.

[68] Chen C, Chen H, Zhang Y, Thomas HR, Frank MH, He Y, Xia R (2020). TBtools: an integrative toolkit developed for interactive analyses of big biological data. Mol Plant.

[69] Dai X, Zhuang Z, Zhao PX (2018). psRNATarget: a plant small RNA target analysis server (2017 release). Nucleic Acids Res.

[70] Shannon P, Markiel A, Ozier O, Baliga NS, Wang JT, Ramage D, Amin N, Schwikowski B, Ideker T (2003). Cytoscape: a software environment for integrated models of biomolecular interaction networks. Genome Res.

[71] Ma S, Wang M, Wu J, Guo W, Chen Y, Li G, Wang Y, Shi W, Xia G, Fu D (2021). WheatOmics: a platform combining multiple omics data to accelerate functional genomics studies in wheat. Mol Plant.

[72] O'Brien KP, Remm M, Sonnhammer EL (2005). Inparanoid: a comprehensive database of eukaryotic orthologs. Nucleic Acids Res.

[73] Yu Y, Ouyang Y, Yao W (2018). shinyCircos: an R/Shiny application for interactive creation of Circos plot. Bioinformatics.

[74] Kumar S, Stecher G, Li M, Knyaz C, Tamura K (2018). MEGA X: Molecular Evolutionary Genetics Analysis across computing platforms. Mol Biol Evol.

[75] Liu YJ, Gao SQ, Tang YM, Gong J, Zhang X, Wang YB, Zhang LP, Sun RW, Zhang Q, Chen ZB (2018). Transcriptome analysis of wheat seedling and spike tissues in the hybrid Jingmai 8 uncovered genes involved in heterosis. Planta.

[76] Livak KJ, Schmittgen TD (2001). Analysis of relative gene expression data using real-time quantitative PCR and the 2(-Delta Delta C(T)) Method. Methods.

[77] Saleh A, Alvarez-Venegas R, Avramova Z (2008). An efficient chromatin immunoprecipitation (ChIP) protocol for studying histone modifications in Arabidopsis plants. Nat Protoc.

