# Supplementary material for: Comprehensive molecular evaluation of the histone methyltransferase gene family and their important roles in two-line hybrid wheat
Source: BMC Plant Biol. 2022 Jun 13;22:290. doi: 10.1186/s12870-022-03639-0 (PMC9190116; doi:10.1186/s12870-022-03639-0)
Supplement: Supplementary file 1 — Additional file 1: Figure S1. (A) and (B): Distribution of two sub-gene families on wheat chromosome. Distribution of two gene families on wheat chromosome. All wheat chromosomes have HKMT gene distribution, which are relatively uniform. However, chromosome 2 and chromosome 3 also tend to cluster. Compared with the HKMT genes, the number of PRMT genes is relatively small, and the distribution of PRMT genes in the whole wheat genome is uneven. In addition, compared with previous research results, most HMT genes have been located in a clear range, and there is only one HKMT gene on the U chromosome, may also indicate that the genome quality of wheat has been greatly improved. Figure S2. Analysis of cis-elements of promoters of TaHMT genes. The 2kb sequence before the CDS of each HMT gene was extracted as the promoter region and submitted to PlantCARE database for cis-regulatory response elements prediction, and the results were visualized by TBtools. Figure S3. Expression analysis all selected TaHMT genes. (A) Expression analysis of 19 genes in spike. (B) Expression analysis of 12 genes in tiller. The relative expression of the female parent of each combination was relative 1, and the expression values for each parent are placed in front of each combination. The horizontal column is the gene name and the vertical column is the name of the samples. Figure S4. The phenotype as well as sampling times at different periods of wheat development. (A) Different periods of wheat spike development and sampling were conducted on this basis at anther separation stage. The length of the red ruler represents 1mm. (B) Tillers from root base to stem about 0.5 cm in length were sampled which contains the apical meristem at about four-leaf stage; P: male parent, M: maternal parent. (C) Phenotypic trait combinations for different tillers at harvest time, from left to right, they were: high parent (HP), middle parent (MP) and low parent (LP). In the picture, the three plants were photogr [file 12870_2022_3639_MOESM1_ESM.pdf]

Additional file 1

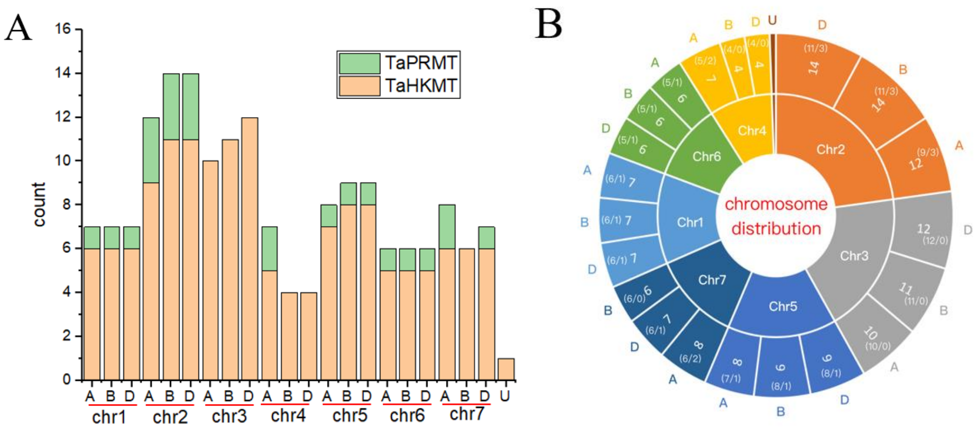

Figure S1 (A) and **(B):** Distribution of two sub-gene families on wheat chromosome. Distribution of two gene families on wheat chromosome. All wheat chromosomes have *HKMT* gene distribution, which are relatively uniform. However, chromosome 2 and chromosome 3 also tend to cluster. Compared with the *HKMT* genes, the number of *PRMT* genes is relatively small, and the distribution of *PRMT* genes in the whole wheat genome is uneven. In addition, compared with previous research results, most *HMT* genes have been located in a clear range, and there is only one *HKMT* gene on the U chromosome, may also indicate that the genome quality of wheat has been greatly improved.

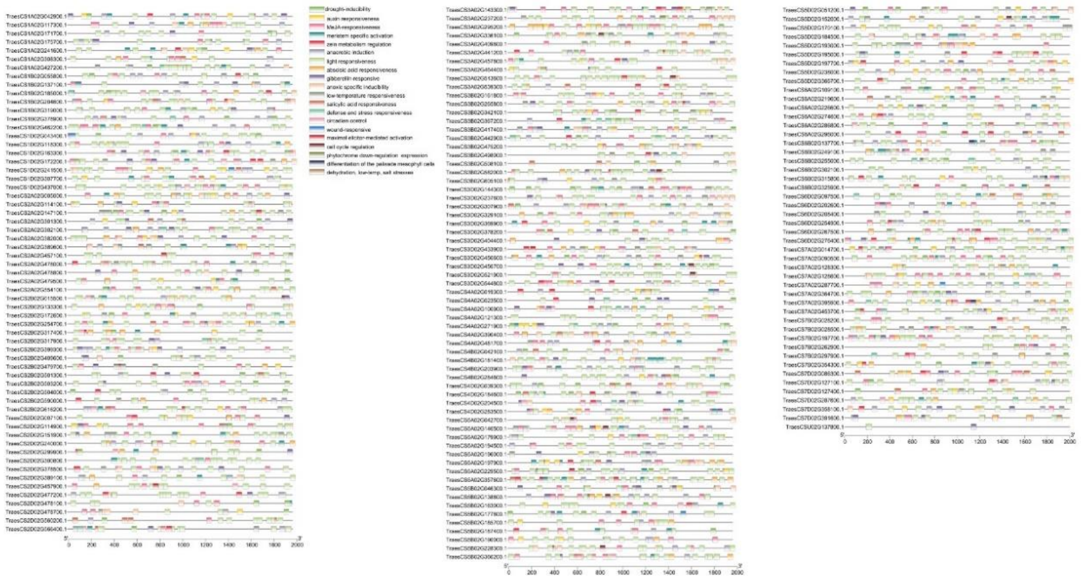

Figure S2 Analysis of cis-elements of promoters of *TaHMT* genes. The 2kb sequence before the CDS of each *HMT* gene was extracted as the promoter region and submitted to PlantCARE database for cis-regulatory response elements prediction, and the results were visualized by TBtools.

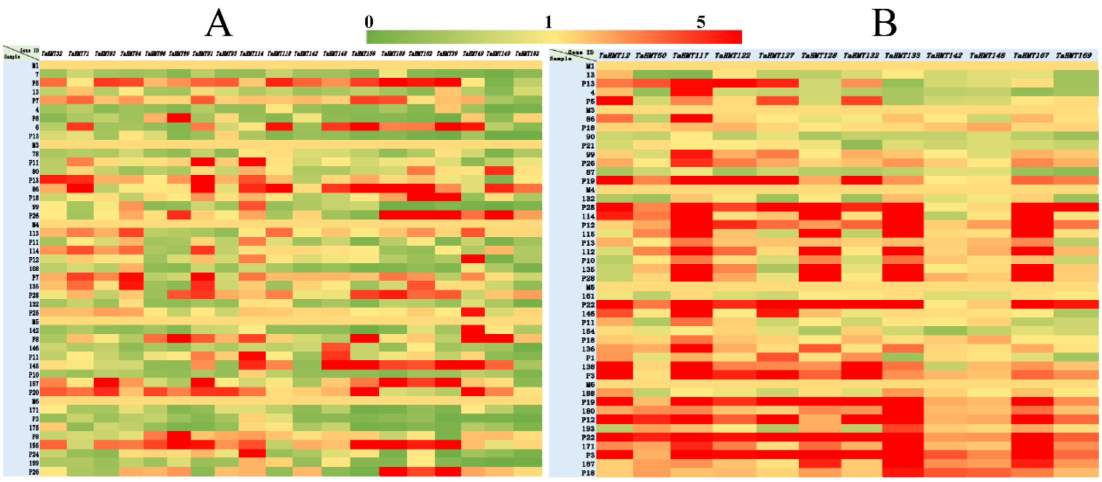

Figure S3 Expression analysis all selected *TaHMT* genes. **(A)** Expression analysis of 19 genes in spike. **(B)** Expression analysis of 12 genes in tiller. The relative expression of the female parent of each combination was relative 1, and the expression values for each parent are placed in front of each combination. The horizontal column is the gene name and the vertical column is the name of the samples.

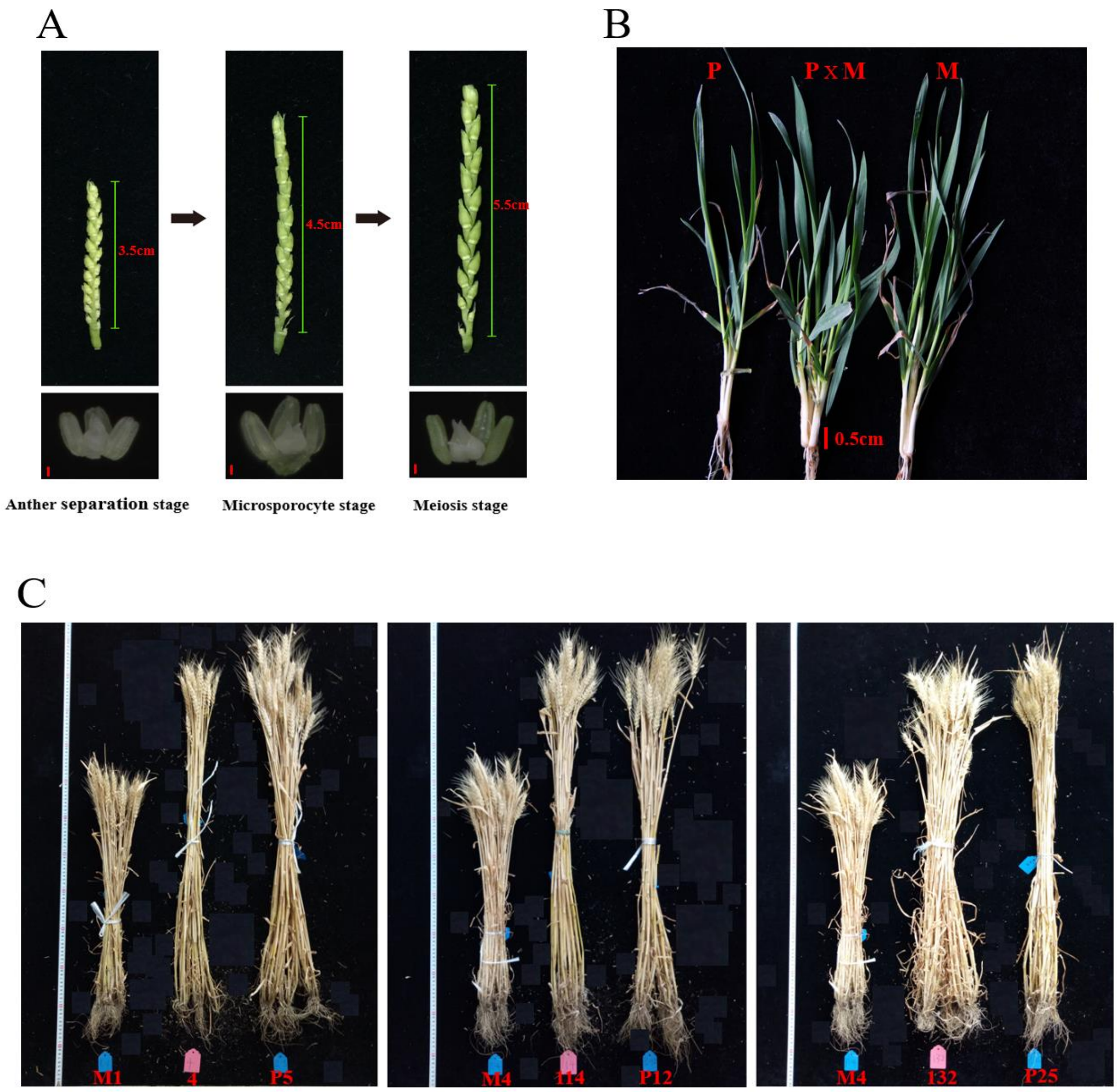

Figure S4 The phenotype as well as sampling times at different periods of wheat development. **(A)** Different periods of wheat spike development and sampling were conducted on this basis at anther separation stage. The length of the red ruler represents 1mm. **(B)** Tillers from root base to stem about 0.5 cm in length were sampled which contains the apical meristem at about four-leaf stage; P: male parent, M: maternal parent. **(C)** Phenotypic trait combinations for different tillers at harvest time, from left to right, they were: high parent (HP), middle parent (MP) and low parent (LP). In the picture, the three plants were photographed as a whole.

A

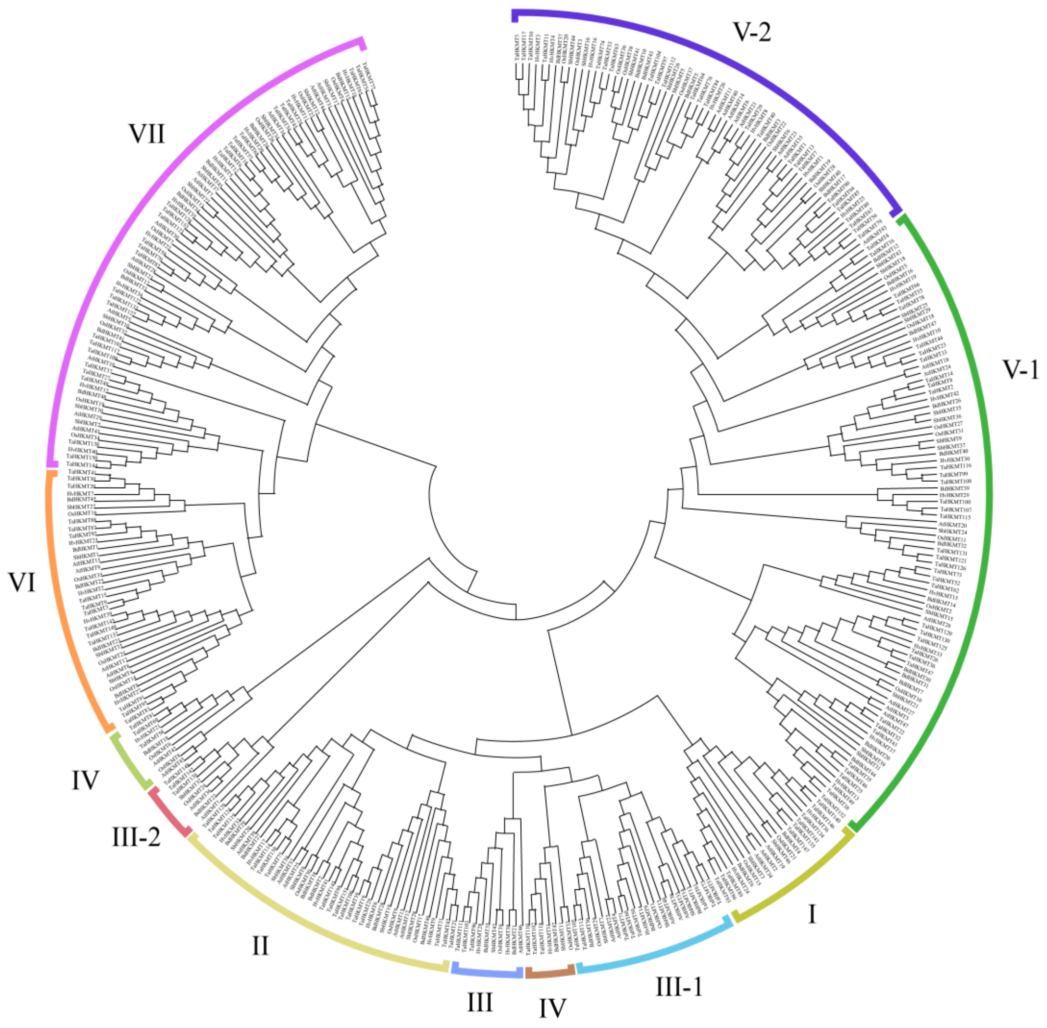

B

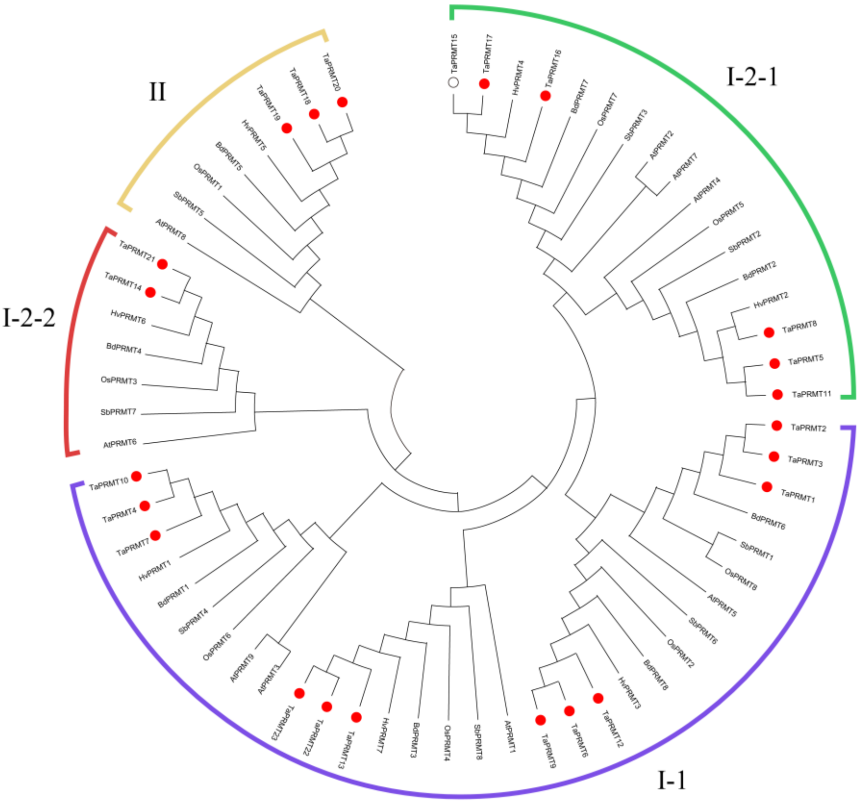

Figure S5 The phylogenetic trees of HKMT **(A)** and PRMT **(B)** proteins from wheat, rice, *Arabidopsis*, *Sorghum bicolor*, *Hordeum vulgare*, and *Brachypodium distachyon* were constructed by MEGA-X using Maximum likelihood method. The HKMT proteins were divided into seven subfamilies and PRMT were divided into four subfamilies based on the previous methods. The members of wheat were marked with red circles in **Fig. S5B**.

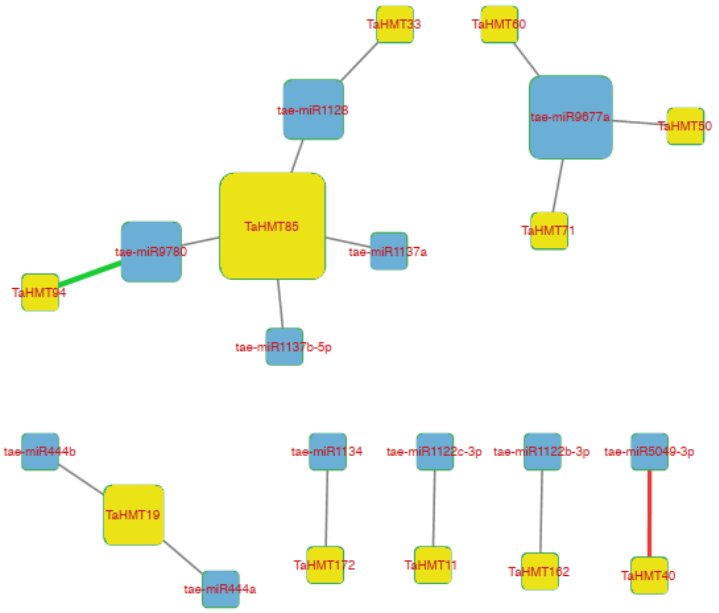

Figure S6 Interaction networks of miRNAs and HMT targets. miRNAs were marked as a blue node and HMT targets were marked as a yellow node. The size of the node represents the number of action sites. The black solid line represents that the mismatch expectation is 3.0, the red thick solid line represents that the mismatch expectation is 1.5, and the green thick solid line represents that the mismatch expectation is 2.5.
